# Life Factors and Melanoma: From the Macroscopic State to the Molecular Mechanism

**DOI:** 10.1002/advs.202501388

**Published:** 2025-10-14

**Authors:** Hanbin Wang, Hengxiang Zhang, Yuqi Zhou, Chunying Li, Weinan Guo

**Affiliations:** ^1^ Department of Dermatology Xijing Hospital Fourth Military Medical University No 127 of West Changle Road Xi'an Shaanxi 710032 China

**Keywords:** environmental factors, genetic factors, life factors, melanoma

## Abstract

Melanoma, a highly aggressive form of skin cancer, is shaped by a complex interplay of genetic, environmental, and lifestyle factors. This review provides a comprehensive synthesis of the macroscopic and molecular mechanisms through which various factors such as gender, age, obesity, smoking, alcohol consumption, diet and nutrients, exercise, ultraviolet (UV) exposure, circadian rhythms, and commonly used medications (e.g., steroids, metformin, non‐steroidal anti‐inflammatory drugs [NSAIDs], and antihistamines), impact melanoma risk, progression, and outcomes. To facilitate a more systematic understanding of melanoma risk and pathogenesis, a comprehensive framework is proposed that delineates these factors into four major categories: intrinsic host characteristics, pharmacological influences, metabolic conditions, and behavioral risk profiles. Within each category, these elements interact at the molecular level, collectively influencing the initiation and progression of melanoma. Furthermore, the translational implications of these findings are evaluated, offering actionable insights for prevention and therapeutic strategies in clinical practice. This review aims to bridge the gap between molecular research and real‐world behavioral determinants, providing a holistic framework that advances our understanding of melanoma pathogenesis and supports the development of improved clinical outcomes.

## Graphical abstract

Melanoma, an aggressive skin cancer, arises from dynamic interactions between genetic, environmental, and lifestyle factors. This review explores how age, gender, obesity, diet, exercise, smoking, alcohol, UV exposure, circadian rhythms, and medications influence melanoma risk and progression. By integrating molecular and epidemiological insights, it reveals key mechanisms driving tumor behavior and highlights modifiable risk factors for prevention and therapy.

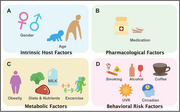

## 1 Background

Melanoma, a highly aggressive and life‐threatening form of skin cancer, arises from the malignant transformation of melanocytes. It is considered the most lethal type of skin cancer, with a steadily increasing incidence over recent decades. Integrating site of origin, molecular profiles, and histological features, the World Health Organization (WHO) Classification of Skin Tumors (4th Edition) categorizes melanoma into four common cutaneous subtypes: superficial spreading melanoma, lentigo maligna melanoma, nodular melanoma, and acral lentiginous melanoma.^[^ ^1^ ^]^ Superficial spreading melanoma, typically found in Caucasians on intermittently sun‐exposed skin, displays radial growth and pagetoid spread of melanocytes. Lentigo maligna melanoma, associated with chronic sun exposure, presents as isolated melanocytes along the dermoepidermal junction and skin appendages. Nodular melanoma lacks a radial growth phase and instead exhibits early vertical proliferation, often forming elevated dermal nodules. Acral lentiginous melanoma, the most common subtype in non‐Caucasian populations, primarily arises on the palms, soles, and nail beds, showing melanocyte proliferation in linear or confluent patterns along the junction. Additional rare subtypes include mucosal melanoma, uveal melanoma, and desmoplastic melanoma.^[^ ^2^ ^]^ Among them, cutaneous melanoma is the most common and is characterized by high metastatic potential and resistance to conventional therapies, making it a leading cause of skin cancer‐related mortality.^[^ ^3^ ^]^ The rising global burden of melanoma highlights the urgent need to identify modifiable risk factors and develop effective strategies for prevention, early detection, and treatment. This underscores the importance of comprehensively understanding the diverse contributors to melanoma pathogenesis and their clinical implications.

**Figure 1:**
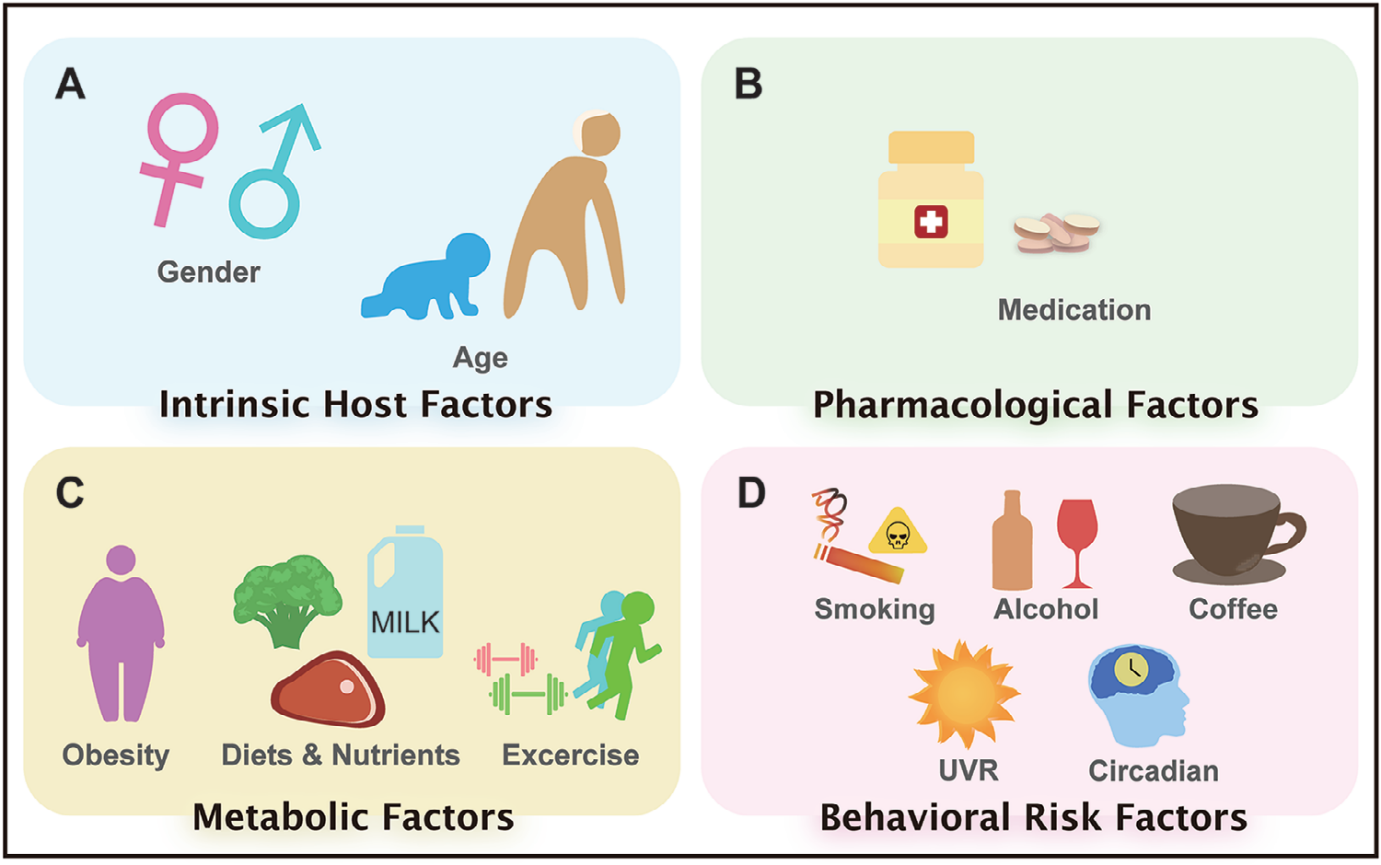
Key factors affecting melanoma pathogenesis. A) Intrinsic host characteristics, including gender, age, and obesity; B) Pharmacological influences; C) Metabolic conditions, including obesity, diets, and nutrients, exercise. D) Behavirol risk profiles, including smoking, alcohol consumption, coffee consumption, UVR exposure, and circadian rhythms.

**Figure 2:**
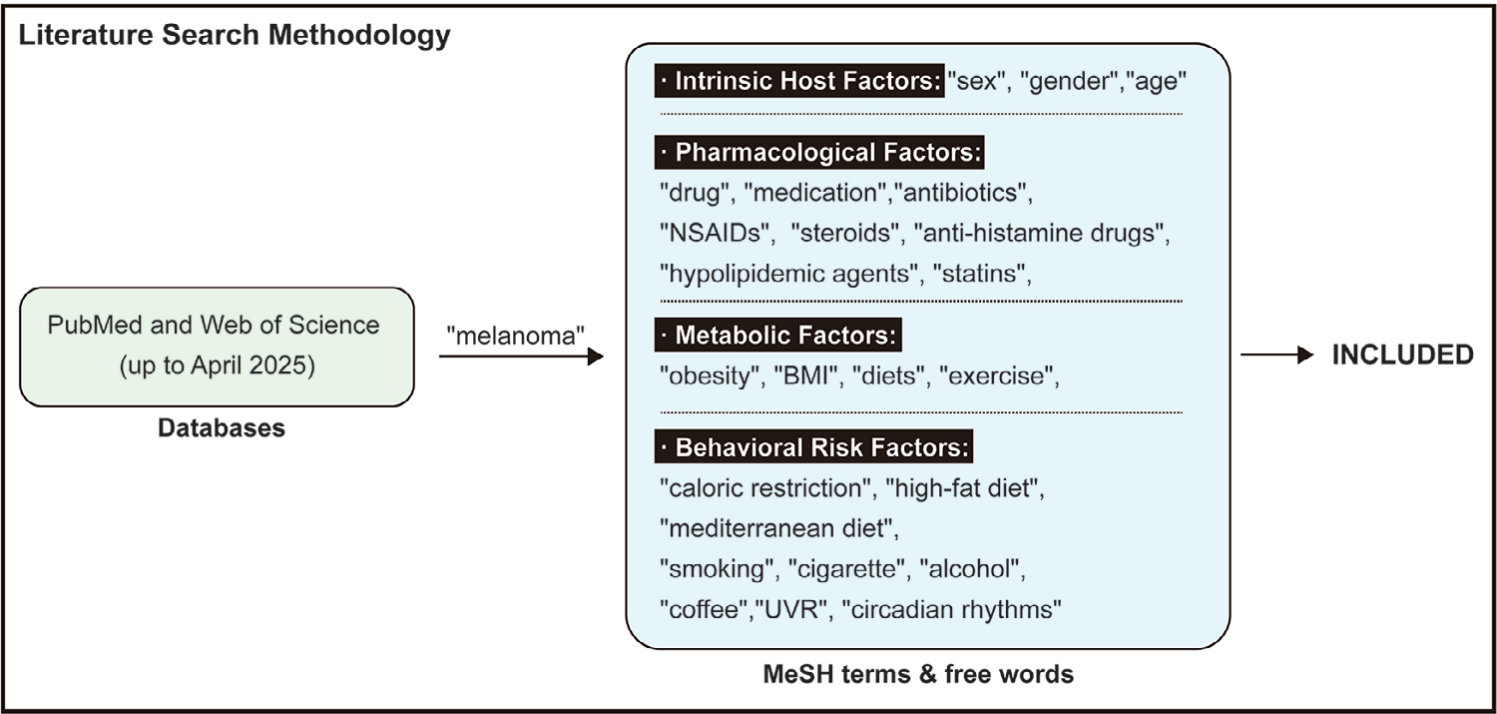
Screening protocol. The following databases were searched electronically up to (April 2025): PubMed and Web of Science. The search strategies were performed through a combination of Mesh terms and free words included (“melanoma” AND (“sex“ OR “gender”)), (“melanoma” AND ”age“), (“melanoma” AND (“drug” OR “medication”)), (“melanoma” AND “antibiotics”), (“melanoma” AND “NSAIDs”), (“melanoma” AND “steroids”), (“melanoma” AND “anti‐histamine drugs”), (“melanoma” AND “hypolipidemic agents“), (“melanoma” AND ” statins“), (“melanoma” AND (”obesity“ OR ”BMI")), (“melanoma” AND (“diets” OR “Caloric Restriction” OR “high‐fat diet” OR “Mediterranean diet”)), (“melanoma” AND (“Smoking” OR “cigarette”)), (“melanoma” AND (“alcohol” OR ”drinking”)), (“melanoma” AND “coffee”), (“melanoma” AND “exercise”), (“melanoma” AND “UVR”), (“melanoma” AND “circadian rhythms”). At the same time, the references cited in the included literature were traced back to ensure that all eligible articles were included.

Lifestyle factors play a significant role in both the development and clinical outcomes of melanoma. Investigating the influence of variables such as gender, age, obesity, smoking, alcohol consumption, coffee intake, dietary patterns, medication, physical exercise, UV exposure, and circadian rhythms allows for a multifactorial exploration of melanoma etiology (**Figure** **1**). This review adopts an integrative perspective by categorizing these factors into four interrelated modules, intrinsic host characteristics, pharmacological influences, metabolic conditions, and behavioral risk profiles, to investigate their collective influence on melanoma. By synthesizing a broad range of clinical and experimental studies, we assess how these factors contribute to melanoma risk and progression, while also examining their roles in immune modulation and therapeutic responsiveness. The screening protocol is shown in **Figure** **2**.

This modular framework, which has rarely been consolidated in prior literature, offers a more unified and mechanistic understanding of melanoma pathogenesis. In the course of this synthesis, the review also identifies notable knowledge gaps and methodological limitations in current research, underscoring the need for more targeted and mechanistic studies. This multidimensional approach bridges clinical observations with foundational biological insights, enabling a more holistic comprehension of melanoma biology. Furthermore, it aligns scientific understanding with real‐world behavioral and clinical contexts, reinforcing the relevance and translational potential of these findings for improving patient outcomes.

## 2 Intrinsic Host Characteristics

### 2.1 The Association between Gender and Melanoma

In general, the incidence and survival rates for cancer vary significantly by sex, with males typically exhibiting lower incidence and survival rates compared to females.^[^ ^4^ ^]^ Factors contributing to these phenomena include differences in sex chromosomal.^[^ ^5^ ^]^ sex‐specific gene regulatory networks,^[^ ^6^ ^]^ levels of sexual hormones, and fat‐free body mass.^[^ ^7^ ^]^ While the influence of gender on tumor incidence and treatment outcomes is well known in various malignancies, such as lung cancer,^[^ ^8^ ^]^ colorectal cancer,^[^ ^9^ ^]^ nasopharyngeal cancer, and glioblastoma,^[^ ^10^ ^]^ its role in melanoma remains an undervalued issue. Therefore, this section aims to elucidate gender‐specific effects on melanoma development and patient prognosis by summarizing current evidence and examining underlying mechanisms.

The gender disparity in melanoma incidence has been extensively studied. It is generally acknowledged that males are at a higher risk of developing melanoma. Among the 425 915 patients diagnosed with melanoma in the United States between 2001 and 2013, the majority were men (56.8%).^[^ ^11^ ^]^ Accounting for differences in their age structure, Garbe et al. extracted age‐standardized incidence rates (ASIR) from melanoma datasets in Denmark, New Zealand, and the Surveillance, Epidemiology, and End Results (SEER) databases of the United States. The 2016 ASIR of men from the United States and New Zealand was significantly higher than that of women. However, there are no such gender differences in the ASIR of Danish patients.^[^ ^12^ ^]^ In China, the incidence of melanoma has also consistently been higher in males across all age groups.^[^ ^13^ ^]^ However, age‐stratified analyses provide further nuance: while males generally exhibit higher incidence overall, younger women, particularly those under 45, show higher incidence rates than age‐matched males.^[^ ^14^ ^]^ In addition, a retrospective analysis of 1279 melanoma patients indicated that, among those under 50, females had a higher incidence of melanoma compared to males.^[^ ^15^ ^]^

Gender also significantly influences the progression and prognosis of melanoma, particularly in terms of mortality and recurrence. Male patients tend to experience higher mortality and recurrence rates than females; this disparity is reported across multiple countries, including Denmark, New Zealand,^[^ ^12^ ^]^ Spain,^[^ ^16^ ^]^ Britain,^[^ ^17^ ^]^ Japan, and others.^[^ ^18^ ^]^ A Swedish study reported that men had a 26% higher risk of mortality and a 19% higher recurrence rate for cutaneous malignant melanoma compared to women.^[^ ^19^ ^]^ In East Asian populations, male mortality rates were 1.51 times higher than those of females.^[^ ^20^ ^]^ Among patients with early‐stage localized cutaneous melanoma, men also exhibit a higher recurrence rate of 35.2% compared to women (21%).^[^ ^21^ ^]^

The increasing use of targeted medication and immune checkpoint inhibitors (ICIs) has revolutionized the treatment of melanoma. Given well‐documented sex differences in immune system function and drug pharmacokinetics⁵, gender may affect therapeutic efficacy.^[^ ^7^ ^]^ In the Netherlands, the objective response rate to systemic therapy in patients with advanced melanoma was lower in males, while female patients exhibited longer progression‐free survival (PFS) and overall survival (OS).^[^ ^22^ ^]^ Furthermore, a study of 563 patients treated with dabrafenib in combination with trametinib found significantly better outcomes in women, with higher hazard ratios (HRs) for both PFS and OS compared to men, suggesting that women are more likely to derive benefit from targeted therapy.^[^ ^23^ ^]^ However, a multicenter retrospective study of 300 *BRAF*‐mutant melanoma patients from Germany and Australia treated with vemurafenib found no statistically significant gender differences in PFS or OS.^[^ ^24^ ^]^ Another study of 315 patients with advanced melanoma suggested that women had lower response rates in a programmed death‐1 (PD‐1) monoclonal antibody model.^[^ ^25^ ^]^ In line with this, men with metastatic melanoma derived significant long‐term benefits from ICI therapy according to a separate cohort study.^[^ ^26^ ^]^

These discrepancies may be attributed to several factors: 1) population heterogeneity across studies, including regional incidences (**Table** **1**) and differences in melanoma subtypes; 2) variation in treatment modalities (e.g., monotherapy vs combination regimens); and 3) limitations related to sample size and age stratification, which may introduce bias. Therefore, the impact of gender on the efficacy of targeted therapies and ICIs warrants further in‐depth investigation.

**Table 1:** Gender‐related melanoma incidence trends in different regions.

Study	PMID	Male	Female	Patients	Continent	Country	Skin color
Yu, 2024	39717039	age >60: Female IR>Male IR, age <60: Female IR<Male IR		4196(in 1990)–16 073(in 2017)	Asia	China	Yellow
Catherine M Olsen 2020	32211827	male to female incidence rate ratios (IRRs)≥1		1279	North America, Europe, Oceania	the US, the UK, Norway, Sweden, Denmark, Australia, and New Zealand	White
Gyrylova, 2014	24716957	IR = 3.49	IR = 3.98		Europe	Russia	White
Duschek, 2013	24184485	ASIR = 9.1	ASIR = 8.4	2 380 109	Europe	Austria	White
Sun, 2025	39343306	161320	141790	303 100	Global	Global 1990–2021	
		ASIR = 4.1	ASIR = 3.16				
Huang, 2023	37296344	ASR = 3.8	ASR = 3.0	324 635	Global	Global 2020	
Helkkula, 2024	39140487	IR = 3.4[2.9‐3.9]	IR = 4.7[4.1‐5.3]	403	Europe	Sweden	White
Dulskas, 2021	33920754	APC = 3.9%	APC = 2.3%	6024	Europe	Lithuania	White
Buja, 2022	35076310	IR = 18.21 [16.76–19.76]	IR = 17.66 [16.15–19.27]	1279	Europe	Italy	White
Garnett, 2016	27021339	IR = 4.6[4.4‐4.8]	IR = 4.0[3.9‐4.1]	6623	North America	USA	93.3%White, 2.7%non‐white, 3.9%unknown
Oliveros, 2025	40054415	IR = 22.66	IR = 17.42	5255	South America	Colombia	
Helgadottir, 2021	32577730	IR = 7.9[9.3 ‐11.2]	IR = 6.4[6.8 – 8.3]	428 793	Europe	Sweden	White
Melo, 2018	30204684	IR = 4.84	IR = 3.22	28 624	South America	Brazil	75% White, 21.9% Brown, 2.4% Black, and 0.7% Yellow/Indigenous

ASR: Age‐Standardized Rate, ASIR: Age‐standardized incidence rates. IR: Incidence Rate. APC: Annual Percentage Change.

**Figure 3:**
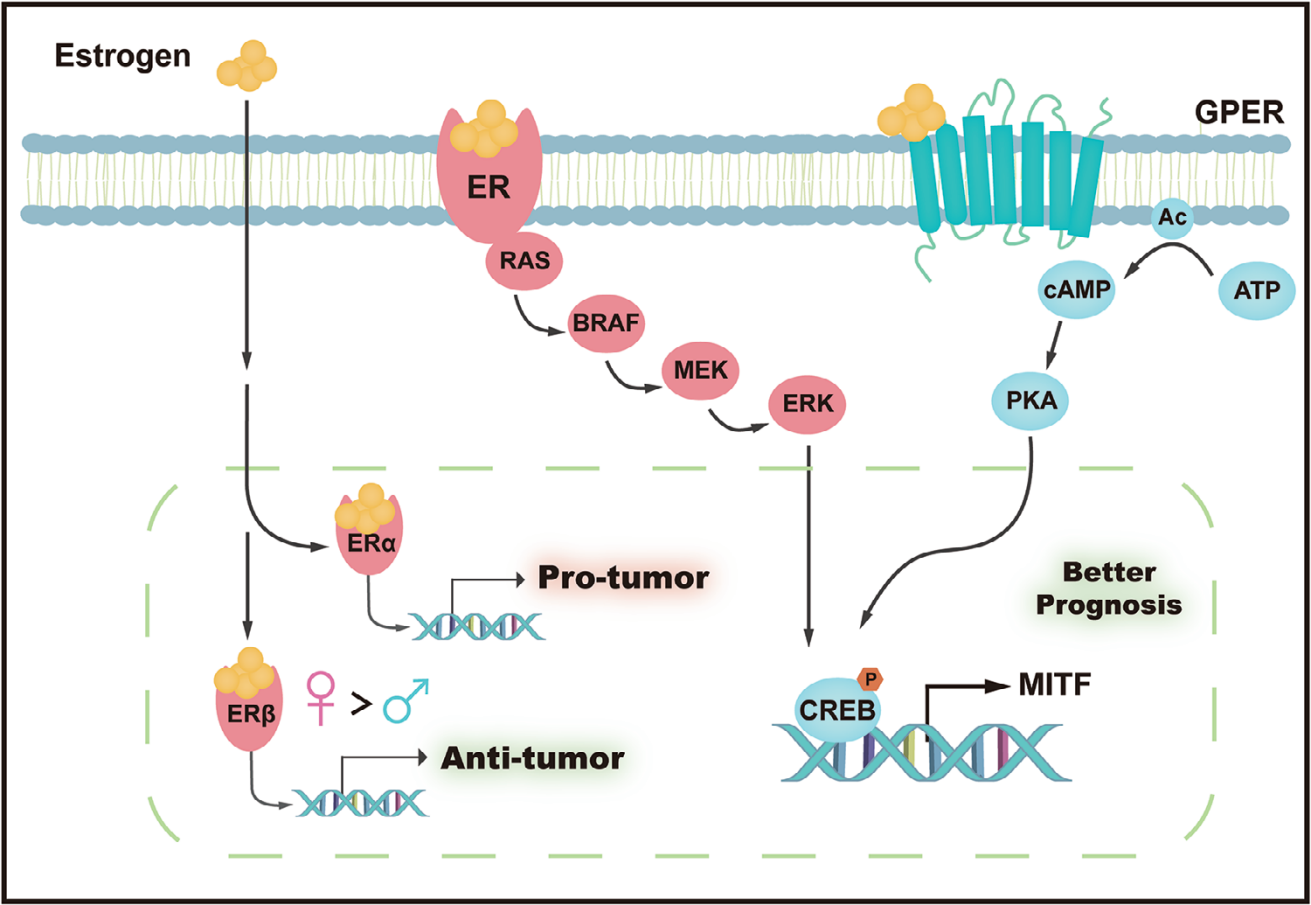
The mechanisms underlying the role of estrogen in melanoma progression. This schematic illustrates the dual roles of estrogen signaling mediated by classical estrogen receptor (ER) and G protein‐coupled estrogen receptor (GPER). Estrogen binds to ERα in nucleus, leading to tumor‐promoting effects. Concurrently, ERβ exhibits anti‐tumor activity, with stronger effects observed in females compared to males. Estrogen binds to ER on cell membrane, activating the RAS‐RAF‐MEK‐ERK signaling cascade. In parallel, GPER activation triggers ATP‐to‐cAMP conversion, activating PKA, which phosphorylates CREB. Phosphorylated CREB induces MITF expression, contributing to better prognosis.

Explorations into the potential underlying mechanisms reveal a complex interplay of exogenous and endogenous factors, encompassing genetic differences, hormonal disparities, variations in the immune system, environmental influences, and social lifestyle factors. Some studies have demonstrated that gender disparities in melanoma might be partially attributed to behavioral differences in men, such as delayed diagnosis and greater sun exposure.^[^ ^17^ ^]^ However, even after adjusting for these factors, males with melanoma exhibit poorer outcomes than females, with sex remaining an independent prognostic factor of survival.^[^ ^27^ ^]^ This suggests the involvement of one or more as‐yet‐unidentified biological mechanisms underlying the observed survival advantage in melanoma based on sex. One such disparity lies in genetic mutations: men tend to harbor a greater number of cell‐division‐associated somatic mutations. Melanomas from males exhibit an overall higher number of missense mutations than those from females, including in genes such as *BRAF* and *NRAS*, even after adjusting for age and clinical covariates.^[^ ^28^ ^]^ Moreover, the greater burden of missense mutations among males leads to the diminished ability to remove mutation‐rich tumor cells and therefore undermines the antitumor immune surveillance, which may explain, in part, the female survival advantage observed clinically.^[^ ^29^ ^]^ Differences in sex hormone levels and receptor signaling also influence immune regulation. Estrogens exert their effects primarily through two receptors, estrogen receptor α (ERα) and estrogen receptor β (ERβ), both of which are members of the nuclear receptor superfamily.^[^ ^30^ ^]^ Additionally, estrogen can elicit a rapid effect that activates non‐nuclear signaling via membrane‐bound receptors, promoting the RAS/BRAF/MEK/ERK axis and increasing phosphorylation of cyclic adenosine monophosphate (cAMP)‐responsive element‐binding protein (CREB).^[^ ^31^ ^]^ Another crucial estrogen receptor is the G protein‐coupled estrogen receptor (GPER), a membrane‐associated receptor that belongs to the G protein‐coupled receptor family. The activation of GPER has been shown to inhibit melanoma cell proliferation, as well as that of prostate and 17β‐estradiol–stimulated breast cancer cells.^[^ ^32^ ^]^ In melanocytes, GPER‐mediated signaling enhances melanogenesis by elevating intracellular cAMP, which in turn activates protein kinase A (PKA) and leads to CREB phosphorylation and upregulation of the microphthalmia‐associated transcription factor (MITF) (**Figure** **3**). Increasing evidence supports a relationship between perturbations of estrogen signaling and tumor initiation, progression, and metastasis. ERα, the predominant estrogen receptor in human epidermis, exerts a role in tumorigenesis by promoting cell proliferation, whereas ERβ appears to exhibit a notable anti‐tumor activity, often acting antagonistically to ERα.^[^ ^33^ ^]^ Although relatively few studies have delved into the mechanistic role of ERα in melanoma, hypermethylation of ERα promoter CpG islands has been implicated in melanoma progression.^[^ ^34^ ^]^ Conversely, ERβ, the predominant ER expressed on melanoma cells,^[^ ^35^ ^]^ has received greater attention. RNA sequencing analysis has indicated that selective ERβ agonists, such as LY500307, can induce interleukin‐1β (IL‐1β) expression in melanoma cells, thereby enhancing antitumor neutrophil recruitment.^[^ ^36^ ^]^ Moreover, the study reveals that extracellular acidosis–induced epithelial‐to‐mesenchymal transition (EMT) in melanoma cells is inversely associated with ERβ expression, shedding light on mechanisms that may underlie the sex‐related disparities frequently observed in cutaneous melanoma.^[^ ^35^ ^]^ Notably, under acidic conditions, melanoma cells from female patients show upregulated ERβ expression at both mRNA and protein levels, whereas those from male patients either do not alter or reduce ERβ expression. Additionally, ERβ is more highly expressed in melanomas from pregnant women and may generally be more abundant in women than in men.^[^ ^37^ ^]^ Intriguingly, recent findings suggest an “obesity paradox” in melanoma: obese men with melanoma exhibit better response to targeted therapy and improved survival rates compared to their normal body mass index (BMI) counterparts.^[^ ^38^ ^]^ This phenomenon may support the protective role of sex hormone/ER activity on disease progression, as obese men typically have higher levels of estrogen in their blood. However, the causal relationship remains unclear, and further investigation is needed.

### 2.2 Aging and Melanoma Pathogenesis

Cancer predominantly affects the elderly population, as aging is associated with a gradual loss of physiological integrity and a decline in immune system function, which in turn increases the risk of tumorigenesis.^[^ ^39^ ^]^ Older individuals are frequently diagnosed at a more advanced stage and may receive less intensive treatment, both of which can negatively affect outcomes.^[^ ^40^ ^]^ Furthermore, their compromised antitumor immunity, which includes reduced numbers of naïve T cells, depletion of tumor‐specific memory T cells, and an increased presence of immunosuppressive cell populations,^[^ ^41^ ^]^ along with other alterations in the tumor microenvironment (TME),^[^ ^42^ ^]^ may further contribute to their poor prognosis. Melanoma is more prevalent in older individuals than in younger adults, and, similar to many cancers, its incidence increases with age.^[^ ^43^ ^]^ According to melanoma patient data from 2001 to 2015 in the U.S. SEER database, the incidence of invasive melanoma decreased among adolescents and young adults between 2006 and 2015, in contrast to a continued rise among older populations.^[^ ^44^ ^]^ In line with this, another study based on data from 84 827 melanoma patients from the Dutch Cancer Registry (1989–2015) revealed a growing disparity in melanoma incidence between age groups, driven by a pronounced increase in incidence of older adults.^[^ ^45^ ^]^ Given a median age of 65 years at diagnosis, with age‐adjusted incidence rates increasing on average by 1.5% per year over the period 2008–2017, melanoma represents a significant tumor burden among the older population.^[^ ^40^ ^]^

In older individuals, melanoma frequently presents with more aggressive histopathological features, including a greater mean Breslow thickness,^[^ ^45^, ^46^ ^]^ higher frequency of ulceration,^[^ ^46^, ^47^ ^]^ and increased mitotic index,^[^ ^48^ ^]^ all of which are associated with worse outcomes. Weiss et al. analyzed cohorts of melanoma patients in three large databases (SEER, the International Melanoma Collaboration Group, and The Cancer Genome Atlas [TCGA]) and found that patients diagnosed after age 65 had the highest overall risk of mortality (adjusted HR 2.19, 95% CI 2.03–2.36).^[^ ^46^ ^]^ Moreover, histopathological subtype distribution varies with age: nodular melanoma, an aggressive subtype with a significantly higher case‐fatality rate, is more prevalent in older patients,^[^ ^49^ ^]^ whereas superficial spreading melanoma, the most common subtype in younger individuals, is less frequently observed in the elderly.^[^ ^50^ ^]^

ICIs represent a promising class of anticancer therapeutics for patients with metastatic melanoma and other malignancies.^[^ ^51^ ^]^ However, ICIs not only enhance immune responses against cancer cells but can also induce inflammatory side effects known as immune‐related adverse events (irAEs).^[^ ^52^ ^]^ These immune‐related toxicities may pose particular challenges in older patients, given their reduced physiological reserve and the presence of age‐related comorbidities. Furthermore, the phenomenon of immunosenescence, which denotes the progressive decline in the functionality of the immune system due to natural aging, may influence both the efficacy and/or toxicity of ICIs.^[^ ^39^ ^]^ Interestingly, immunosenescence does not necessarily imply a poor prognosis for immunotherapy. A multicenter, international retrospective study of 928 geriatric patients (aged ≥80 years) with various cancers treated with single‐agent ICIs found no significant differences in clinical outcomes between those aged <85 years and those ≥85 years.^[^ ^53^ ^]^ Similarly, Ben‐Betzalel et al. retrospectively analyzed 144 patients with metastatic melanoma who received ICI therapy and reported comparable PFS and OS in patients aged 65–80 and 80–100 years.^[^ ^54^ ^]^ Moreover, in another study involving 254 patients across a broader age range, 57 (22.4%) were <50 years old, 85 (33.5%) were age 50–64, 65 (25.6%) were age 65–74, and 47 (18.5%) were ≥75 years, researchers demonstrated that patients with melanoma safely tolerate immunotherapy and effective across all age groups.^[^ ^55^ ^]^ Unexpectedly, several studies have shown that older adults may derive even greater benefit from immunotherapy. Among 655 melanoma patients treated with pembrolizumab, higher complete response rates were observed in patients over 65 years of age.^[^ ^56^ ^]^ This advantage extended beyond response rates to improved prognosis. A multicenter study involving 538 melanoma patients treated with pembrolizumab revealed that for every 10‐year increase in age, the risk of disease progression decreased by 13%, with patients over 60 years of age demonstrating higher efficacy with anti‐PD‐1 therapy.^[^ ^57^ ^]^ The clinical outcomes are consistent with the trait. In a single‐center cohort analysis, patients older >65 years treated with immunotherapy exhibited a superior mean PFS (4.8 vs 3.4 months; *p* = 0.04) and OS (not reached vs 10.1 months; *p* = 0.009) than younger patients in univariate analysis.^[^ ^58^ ^]^ Similar findings have been reported in studies that used age cutoffs of 60 or 70 years, respectively.^[^ ^59^, ^60^ ^]^ However, some studies have suggested the opposite,^[^ ^61^ ^]^ with the younger group (20–59 years) having significantly better survival. Notably, such conclusions are difficult to apply in clinical practice for predicting patients' immunotherapy prognosis,^[^ ^62^ ^]^ as age is a continuous variable, while the conclusions drawn from various studies are based on a binary classification of age. To address this issue, predictive models based on machine learning algorithms and nomograms may offer a more nuanced understanding of age‐related effects on immunotherapy outcomes, since nomograms derived from LASSO or Cox regression analyses have already been applied successfully in predicting tumor recurrence^[^ ^63^ ^]^ and immunotherapy responses.^[^ ^64^ ^]^ Furthermore, incorporating aging‐related signatures, including the composition and functional status of tumor‐infiltrating immune cells (TIICs)^[^ ^65^ ^]^ and genomic mutation burden,^[^ ^66^ ^]^ into predictive models could further enhance their accuracy and clinical applicability.

The high incidence and poor prognosis of melanoma in elderly patients may be attributed to both endogenous and exogenous factors. First, melanoma in older adults is frequently linked to clinical manifestations of chronic, cumulative sun exposure, including solar elastosis, commonly referred to as photoaging,^[^ ^67^, ^68^ ^]^ Second, diagnostic delays are common in this population due to several reasons: difficulties in performing self‐skin examinations owing to visual impairments or physical limitations, absence of a partner to assist with home examinations,^[^ ^47^ ^]^ or lower socioeconomic status, all of which contribute to delayed diagnoses and ultimately poorer clinical outcomes.^[^ ^45^, ^69^ ^]^ At the micro‐level, both molecular characteristics and the composition of the TME contribute to age‐related melanoma behavior.

Molecularly, aging is associated with an increased frequency of *NRAS* mutations and a decreased frequency of *BRAF* mutations, indicating divergent age‐dependent oncogenic pathways. A mathematical model based on the TCGA database has revealed that mutations caused by ultraviolet radiation (UVR) and cell division gradually increased with patients’ age.^[^ ^28^ ^]^ For example, an increased frequency of *NRAS* mutations is observed with advancing age, and these mutations are linked to poorer outcomes.^[^ ^67^, ^70^, ^71^ ^]^. However, *BRAF* mutation, a molecular hallmark in approximately 50% of primary melanomas, is inversely proportional to age and solar elastosis.^[^ ^72^ ^]^ This mutation does not influence the disease‐free interval following the initial diagnosis, but it does indicate a trend towards a worse prognosis in stage 4 disease.^[^ ^73^, ^74^ ^]^ Moreover, younger patients with metastatic melanoma tend to harbor a high prevalence of *BRAF* mutations with a predominance of the V600E genotype,^[^ ^70^, ^72^, ^75^ ^]^ while older patients exhibit a lower prevalence of *BRAF* mutations overall, with a predominance of non‐V600E genotypes, such as V600K.^[^ ^70^, ^72^, ^75^ ^]^ Within the aged TME, fibroblasts play a pivotal role. The expression of hyaluronan and proteoglycan link protein 1 (HAPLN1), secreted by fibroblasts and essential for maintaining extracellular matrix (ECM) integrity, is significantly reduced in aged skin. This reduction impairs the infiltration of both CD4⁺ and CD8⁺ T cells, thereby fostering an immunosuppressive environment conducive to melanoma progression.^[^ ^76^ ^]^ Moreover, diminished HAPLN1 increases the permeability of lymphatic endothelial cells (LECs), promoting distant metastasis over lymph node involvement.^[^ ^77^ ^]^ Besides, aged fibroblasts produce more secreted frizzled‐related protein 2 (sFRP2), a Wnt signaling antagonist that enhances both angiogenesis and metastasis of melanoma. sFRP2 also triggers a multi‐step signaling cascade in melanoma cells, leading to a reduction in β‐catenin and MITF, and consequently, the loss of a key redox effector, apurinic/apyrimidinic endonuclease 1 (APE1). The loss of APE1 diminishes the melanoma cells’ ability to repair reactive oxygen species (ROS)‐induced DNA damage, thereby increasing their resistance to targeted therapy, such as vemurafenib.^[^ ^78^ ^]^ Aging also influences melanoma progression by modulating lipid metabolism within the TME. When exposed to the lipid secretome of aged fibroblasts or cocultured with them, melanoma cells exhibit increased lipid uptake via the fatty acid transport protein 2 (FATP2), which is upregulated in melanoma cells within the aged TME and is known to play a role in lipid synthesis and accumulation.^[^ ^79^ ^]^ In parallel, aged fibroblasts secrete insulin‐like growth factor binding protein 2 (IGFBP2), which triggers the upregulation of the phosphoinositide 3‐kinase (PI3K)‐dependent fatty acid biosynthesis program in melanoma cells, thereby enhancing tumor invasiveness.^[^ ^80^ ^]^

In the context of immunotherapy, although various studies have indicated that older adults often exhibit comparable efficacy to younger populations, investigations into the underlying mechanisms remain limited. Researchers found that the low frequency of naïve CD8^+^ T cells, elevated levels of osteoprotegerin and monocyte chemoattractant protein 4 (MCP‐4), and a higher ratio of CD8^+^ T cells to regulatory T cells (Tregs) in the tumors of elderly mice might be responsible for improved immunotherapy response.^[^ ^57^ ^]^ Nonetheless, preliminary clinical data are encouraging, suggesting that older adults may benefit from ICIs without experiencing increased toxicity. These findings underscore the need for more in‐depth studies to elucidate how age influences immunotherapy responses. However, preliminary data on immunotherapies are encouraging, suggesting that older adults may benefit from the ICI revolution in oncology without increased toxicity. These findings underscore the need for more in‐depth studies to elucidate how age influences immunotherapy responses.

Sex and age jointly influence melanoma development through genetic alterations, hormonal regulation, and immune microenvironment dynamics (Table S1, Supporting Information). Genetically, male melanoma patients exhibit higher mutation rates in *BRAF* and *NRAS*, along with lower expression of ERβ, while elderly patients tend to have increased *NRAS* mutations and decreased *BRAF* mutations. This suggests that, although elderly males constitute a high‐risk demographic, their oncogenic drivers may differ from those in other groups. Hormonally, estrogen suppresses tumorigenesis through the GPER/cAMP/MITF pathway, and the higher estrogen levels and ERβ expression in females suggest that elevated estrogen in obese males may partially contribute to their improved prognosis. Immunologically, estrogen enhances antitumor immunity by promoting IL‐1β secretion and neutrophil recruitment through ERβ activation. Given that 55 years of age is conventionally recognized as the threshold for menopause, advancing age may influence tumor progression via estrogen‐related pathways. Between 1990 and 2021, the age‐standardized prevalence rate (ASPR) and age‐standardized mortality rate (ASMR) of malignant melanoma in premenopausal women were consistently lower than those in postmenopausal women.^[^ ^81^ ^]^

Although aging is associated with immunosenescence, an elevated CD8^+^ T cell/Treg ratio in the tumor microenvironment of elderly patients may potentiate responsiveness to immunotherapy. In summary, elderly male patients exhibit both a higher incidence of melanoma and poorer clinical outcomes, highlighting the need to prioritize preventive strategies such as minimizing UV exposure and undergoing regular dermatological screening to assess pigmented lesions for potential excision, thereby mitigating cumulative carcinogenic risks. Current age‐stratified studies, however, rely on binary classifications that fail to accurately capture the nuanced impact of age on immunotherapy outcomes. Instead, machine learning‐based predictive models and nomogram visualizations are warranted for precise stratification. Such predictive tools could facilitate personalized treatment selection by integrating individual biological profiles, ultimately optimizing therapeutic efficacy while minimizing adverse outcomes in clinical practice. This integrated approach addresses both prevention and precision medicine needs in the management of melanoma among elderly male patients. Notably, conventional high‐risk features, such as elderly male status, should be re‐evaluated using molecular profiling to enable individualized risk assessment. For instance, the most up‐to‐date research reveals that male aging drives a uniquely aggressive and therapy‐resistant TME through fibroblast‐mediated AXL/BMP2 signaling.^[^ ^82^ ^]^ By highlighting the combined effects of sex and age on melanoma, these findings underscore the importance of integrating multi‐omics data in future research to unravel the molecular mechanisms underlying these complex interactions.

## 3 Pharmacological Influences

As previously discussed, melanoma is more prevalent and severe in older individuals than in younger counterparts, along with immune dysfunction,^[^ ^83^ ^]^ infectious diseases,^[^ ^84^ ^]^ diabetes mellitus,^[^ ^85^ ^]^ and cardiovascular disease.^[^ ^86^ ^]^ Adherence to complex medication regimens, including steroids, antihistamines, NSAIDs, hypoglycemic agents (e.g., metformin), hypolipidemic agents (e.g., statins), and antibiotics, poses significant challenges for melanoma patients, with these pharmacological agents exerting varying effects on melanoma (**Table** **2**).

**Table 2:** Medication and Melanoma Pathogenesis.

Medication	Effect on incidence	Effect on prognosis	Impact on immunotherapy
Steroids	Not reported	51%‐126% higher ACM;^[^ ^89^ ^]^ Increase tumor burden;^[^ ^96^ ^]^ Compromise tumor‐killing of TILs.^[^ ^95^ ^]^	Inferior PFS and OS 54% patients developed irAEs.^[^ ^90^ ^]^
Antihistamines	Not reported	HRH1: enhancing melanoma growth.^[^ ^98^ ^]^ HRH4: significant in vivo antitumor effect.^[^ ^102^ ^]^	More than tripled objective response rate to anti‐PD‐1 treatment.^[^ ^99^ ^]^
NSAIDs (Aspirin)	Reduces melanoma risk (12‐20%);^[^ ^105^ ^]^ Reduced melanoma risk in females (adjusted OR: 0.54);^[^ ^108^ ^]^ Increased risk in males(adjusted RR: 1.83).^[^ ^110^ ^]^	Better survival in stage II and III melanoma;^[^ ^114^ ^]^ Lower Breslow thickness((95% CI: 0.0297‐0.8127).^[^ ^115^ ^]^	Enhance immune responses and the effectiveness of ICI therapy;^[^ ^120^ ^]^ Enhance immune responses when combined with ICI.^[^ ^123^ ^]^
Metformin	Modest reduction in melanoma risk(OR: 0.82).^[^ ^129^ ^]^	Improves ACS(HR = 0.68), OS(HR = 0.59,0.90.0.98)^*^;^[^ ^130^ ^]^ Enhances tumor immune response, apoptosis, and inhibits metastasis.^[^ ^134^ ^]^	Enhance the effectiveness of immunotherapy by increasing TILs (CD4+, CD8+); reduce exhausted T cells, and improving tumor‐associated macrophage polarization.^[^ ^137^ ^]^ Improve immune surveillance.^[^ ^138^ ^]^
Antibiotics	Not reported	Promotes distal tumor progression.^[^ ^165^ ^]^	Impair the efficacy of immunotherapy.^[^ ^158^ ^]^ Reduces efficacy of ICI and small‐molecule TKIs;^[^ ^163^ ^]^ Associated with higher incidence of immune‐mediated colitis and lower response rates;^[^ ^159^ ^]^ Leads to reduced T cell activation and effectiveness of ICI.^[^ ^164^ ^]^
Cholesterol Modulators (e.g., Lovastatin, Simvastatin, PCSK9 Inhibitors)	Reduce melanoma incidence: lovastatin (OR = 0.52); fibrate (OR = 0.58).^[^ ^140^, ^375^ ^]^	Associated with improved OS(HR = 0.38),^[^ ^141^ ^]^ reduced recurrence(adjusted HR = 0.55);^[^ ^142^ ^]^ The combination of statins and other antitumor agents(dacarbazine) shows considerable promise;^[^ ^151^ ^]^ Low cholesterol levels inhibit T cell proliferation and induce autophagy‐mediated apoptosis.^[^ ^153^ ^]^	PCSK9 inhibitors improve ICI efficacy by promoting immune cell activation and reducing tumor‐induced immune suppression.^[^ ^154^ ^]^

ACM: All‐caused Mortality, irAEs: Immune‐related Adverse Events, HRH1:histamine receptor H1, HRH4: histamine receptor H4, PFS: Progression‐Free Survival, OS: Overall Survival, CI: Confidence Interval, ICI: Immune Checkpoint Inhibitor, TILs: Tumor‐Infiltrating Lymphocytes, TKIs: Tyrosine Kinase Inhibitors, OR: Odds Ratio, HR: Hazard Ratio.

* The three OS values correspond to 24–30 months of post‐diagnostic use, pre‐diagnostic use, 0–6 months of post‐diagnostic use, respectively.

### 3.1 Steroids

Corticosteroids are frequently employed in melanoma treatment to manage the irAEs associated with ICIs and targeted therapies.^[^ ^87^, ^88^ ^]^ However, their use has been linked to adverse clinical outcomes and increased tumor burden. Numerous studies have shown that the use of steroid administration may negatively impact melanoma prognosis. For instance, data from the SEER database demonstrated that, compared with no steroid use, recent steroid exposure (within ≤1 month and 1–3 months prior to ICI initiation) was associated with a 126% and 51% increase in all‐cause mortality (ACM) within 3 months post‐ICI initiation, respectively.^[^ ^89^ ^]^ In a multicenter retrospective study, early‐onset irAE (within 8 weeks of anti‐PD‐1 initiation) with high‐dose corticosteroids use (≥60 mg prednisone equivalent daily) was independently associated with worse post‐irAE PFS/OS.^[^ ^90^ ^]^ To isolate the impact of glucocorticoids (GCs) dosage, another study investigated a distinct cohort of patients with the same irAEs who received different GC doses. It found that higher doses were correlated with reduced survival, suggesting a dose‐dependent negative effect on ICI efficacy.^[^ ^91^ ^]^ Optimal GC dosing strategies, including low‐dose administration (<60 mg prednisone equivalent daily) or gradual dose escalation, may potentially improve clinical outcomes. Moreover, emerging evidence suggests that Janus kinase inhibitors (JAKi, e.g., tofacitinib) represent a promising alternative for managing irAEs, as demonstrated by their protective effects in cancer patients experiencing irAEs.^[^ ^92^ ^]^ Mechanistically, JAKi may enhance anti‐PD‐1 immunotherapy efficacy through modulation of T‐cell function^[^ ^93^ ^]^ and dynamic regulation of myeloid cell differentiation.^[^ ^94^ ^]^ Nevertheless, these findings require further validation through large‐scale clinical trials to confirm their therapeutic potential and safety profile.

The precise mechanisms by which steroids affect patient survival remain unclear. In vitro studies indicate that dexamethasone, administered at both low or intermediate/high doses, compromises the activation and tumor‐killing capacity of CD8^+^ tumor‐infiltrating lymphocytes (TILs).^[^ ^95^ ^]^ Moreover, dexamethasone significantly increased the expression and activity of Rho‐associated kinase 1/2 (ROCK1/2). Notably, Rho signaling via ROCK has previously been shown to activate the PI3K/ protein kinase B (Akt) pathway, thereby promoting melanoma progression.^[^ ^96^ ^]^ These findings indicate that ROCK1/2 may mediate the pro‐metastasis effects of GCs.^[^ ^97^ ^]^ As a crucial intervention for irAEs, further investigation into their potential contributions and mechanisms to melanoma metastasis is required.

### 3.2 Antihistamines

The reinvigoration of antitumor immunity remains an ongoing challenge, particularly in individuals with pre‐existing immune dysfunction. In tumor‐bearing mice, histamine was found to promote melanoma growth primarily through histamine receptor H1 (HRH1), rather than histamine receptor H2 (HRH2). Activation of HRH1 suppresses the transcription of tumor suppressor genes such as insulin‐like growth factor II receptor (*IGF‐IIR*) and the anti‐angiogenic matrix protein fibulin‐5 (*FBLN5*), leading to decreased intracellular protein levels and reduced availability in the plasma membrane and extracellular matrix, respectively.^[^ ^98^ ^]^ Moreover, clinical evidence indicates that pre‐existing allergic conditions or high histamine levels can dampen immunotherapy responses, conversely, cancer patients who took antihistamines during immunotherapy had significantly improved survival outcomes. Mechanistically, HRH1‐activated macrophages polarize toward an M2‐like immunosuppressive phenotype, characterized by increased expression of the immune checkpoint VISTA (V‐domain Ig suppressor of T cell activation), which induces T cell dysfunction. HRH1 knockout or antihistamine treatment reversed macrophage‐mediated immunosuppression, restored T cell cytotoxic activity, and enhanced immunotherapy efficacy.^[^ ^99^ ^]^ In addition, recent studies have highlighted a novel role of histamine receptor H4 (HRH4) in cancer progression, identifying it as a promising molecular target for anticancer drug development,^[^ ^100^ ^]^ including in melanoma.^[^ ^101^ ^]^ Preclinical data further provided evidence that HRH4 agonists generate significant in vivo antitumor effects,^[^ ^102^ ^]^ although further studies are needed to elucidate their therapeutic potential.

### 3.3 NSAIDs

Acetylsalicylic acid (ASA) has become one of the most commonly used drugs, given its role as an analgesic, antipyretic, and agent for cardiovascular prophylaxis.^[^ ^103^ ^]^ Long‐term ASA use has been shown to reduce the risk of colorectal cancer.^[^ ^104^ ^]^ For melanoma, several clinical trials have reported that NSAIDs may lower the risk of disease development. One study demonstrated that regular use of low‐dose ASA for over 5 years reduced melanoma risk by 12%, with higher doses associated with risk reduction exceeding 20%.^[^ ^105^ ^]^ In a case‐control study, researchers demonstrated that patients who used NSAIDs had a 13% lower risk of melanoma than those who did not. This effect was particularly pronounced among long‐term users (≥7 years) and high‐intensity users (>25% prescription coverage during the total duration of use).^[^ ^106^ ^]^

However, the protective effects of NSAIDs appear to be sex‐specific. The ASA in Reducing Events in the Elderly study found ASA reduced melanoma incidence in women but showed no significant effect in men.^[^ ^107^ ^]^ Supporting studies further demonstrated that low‐dose ASA significantly lowered melanoma risk in women (OR = 0.54) but not in men.^[^ ^108^ ^]^ Moreover, in a cohort of 59 806 postmenopausal women, ASA use was associated with a 21% reduction in melanoma risk. Notably, the protective effect increased with longer duration of use: women who took ASA for more than five years experienced a 30% reduction in melanoma incidence. However, the use of non‐ASA NSAIDs and acetaminophen did not yield significant effects.^[^ ^109^ ^]^ In contrast, Orrell et al. found that ASA use increased the overall incidence of melanoma, with this risk elevation confined to male patients.^[^ ^110^ ^]^ While the impact of NSAIDs on melanoma remains controversial, their gender‐specific effects on outcomes have been consistently observed across multiple studies. The underlying reasons remain unclear but may involve differences in pharmacokinetics and pharmacodynamics between sexes, especially in platelet function, where women achieve higher plasma concentrations at equivalent doses.^[^ ^111^ ^]^ Additionally, since NSAIDs may modulate oxidative stress, sex‐specific variations in antioxidant enzyme levels may also contribute to observed disparities.^[^ ^112^ ^]^ These findings underscore the importance of considering gender as a biological variable in melanoma research and warrant further investigation into the underlying mechanisms.^[^ ^113^ ^]^

NSAIDs may improve the prognosis of melanoma patients. In a retrospective analysis of 1522 individuals, post‐diagnostic ASA use was found to reduce mortality in patients with stage II and stage III melanoma.^[^ ^114^ ^]^ In addition, ASA users also had significantly lower Breslow thickness compared to non‐users.^[^ ^115^ ^]^ However, the association between NSAID use and clinical outcomes in patients receiving immunotherapy remains inconclusive. A retrospective analysis of 159 metastatic melanoma patients treated with ipilimumab showed no significant association between NSAID use and OS.^[^ ^116^ ^]^ Similarly, a larger multicenter retrospective study encompassing 1012 patients with non‐small cell lung cancer, melanoma, and renal cell carcinoma receiving ICIs found no OS benefit with NSAID co‐administration.^[^ ^117^ ^]^ These findings were further supported by a comprehensive meta‐analysis, demonstrating that NSAID use did not significantly affect clinical outcomes in patients undergoing ICI therapy across various cancer types, including overall response rate, PFS, or OS.^[^ ^118^ ^]^ Notably, a phase III clinical trial evaluating the prognostic effects of NSAIDs combined with pembrolizumab in melanoma patients also reported that while NSAID use was not associated with treatment efficacy, it had no significant effect on the risk of irAEs.^[^ ^119^ ^]^ In conclusion, the observed discrepancies across studies may be attributed to variations in NSAID type, dosage, timing, and frequency. Therefore, more rigorously controlled clinical studies are needed to clarify the role of NSAIDs in melanoma treatment.

Numerous studies have demonstrated the mechanisms through which NSAIDs reduce melanoma incidence and improve patient prognosis, elucidating their cellular and molecular effects. NSAIDs are widely recognized to impair prostanoid biosynthesis by irreversibly inhibiting specific or all cyclooxygenase (COX) isoforms. Inhibition of the COX‐1/thromboxane A2 (TXA2) pathway in platelets leads to decreased platelet aggregation on tumor cells, reduced endothelial activation, diminished adhesion of B16F10 melanoma cells to the endothelium, and impaired recruitment of metastasis‐promoting monocytes and macrophages, collectively limiting the establishment of a premetastatic niche.^[^ ^120^ ^]^ ASA inhibition of COX‐2 may also halt melanoma progression, as COX‐2 is often overexpressed in melanoma cells and associated with disease advancement.^[^ ^121^ ^]^ In cells, ASA is transformed into prostaglandin E2 (PGE2) via COX enzymes and terminal prostaglandin E synthases (PGES), and PGE2 has been reported to enhance melanomagenesis and tumor progression.^[^ ^122^ ^]^ Zelenay et al. demonstrated that tumor‐derived PGE2 directly induces myeloid cells to produce cancer‐promoting factors, including interleukin‐6 (IL‐6), C‐X‐C motif chemokine ligand 1 (CXCL1), and granulocyte colony‐stimulating factor (G‐CSF). This reprograms the TME from a cytotoxic milieu to one that facilitates tumor growth. They also revealed that COX inhibitors enhance the efficacy of anti‐PD‐1 immunotherapy.^[^ ^123^ ^]^ The AXL receptor tyrosine kinase (AXL), a downstream product of PGE2, its high expression is implicated in drug resistance, EMT, and enhanced survival of melanoma stem cells.^[^ ^124^, ^125^, ^126^ ^]^ Moreover, NSAIDs have been shown to downregulate PKA activity via the PGE2/EP2/cAMP/PKA signaling pathway. This disruption interferes with the PKA‐dependent interaction between cell division cycle 37 (CDC37) and heat shock protein 90 (HSP90), leading to improper folding and subsequent degradation of AXL.^[^ ^127^ ^]^ Additionally, NSAIDs suppress nuclear factor‐kappa B (NF‐κB), a well‐known anti‐apoptotic transcription factor implicated in melanoma progression.^[^ ^128^ ^]^

### 3.4 Metformin

Metformin, a widely used biguanide hypoglycemic medication, has recently been shown to possess tumor‐suppressive properties, supported by growing evidence. Meta‐analyses of both randomized controlled trials (RCTs) and cohort studies have found no statistically significant association between metformin use and skin cancer risk. Nevertheless, some studies suggest a modest reduction in skin cancer incidence among metformin users.^[^ ^129^ ^]^ In melanoma, metformin has been associated with improved prognosis. In patients with both melanoma and diabetes, metformin use correlated with enhanced OS, regardless of the timing of administration (pre‐, post‐, or peri‐diagnostic), and exhibited a dose‐response relationship.^[^ ^130^ ^]^ An analysis of 4790 diabetic melanoma patients reported a significantly lower 5‐year recurrence rate among metformin users.^[^ ^131^ ^]^ However, clinical evidence remains inconclusive: a small open‐label trial (*n* = 17) observed no therapeutic benefit of high‐dose metformin (1g TID) following progression on first‐line melanoma therapy.^[^ ^132^ ^]^ Similarly, a retrospective study of checkpoint inhibitor‐treated patients (*n* = 55) showed a non‐significant trend toward improved OS with concurrent metformin use.^[^ ^133^ ^]^ In contrast, a larger cohort study (*n* = 1162) confirmed prolonged OS among diabetic patients with stage I–IV melanoma, although no improvement in melanoma‐specific survival was observed.^[^ ^130^ ^]^ These findings further suggest that metformin may exert indirect survival benefits through pleiotropic mechanisms, including metabolic modulation, improved glycemic control and insulin sensitivity, attenuation of oxidative stress, and cardiovascular protection.

Preclinical studies have demonstrated that metformin induces tumor cell apoptosis and enhances anti‐tumor immunity. It significantly suppresses melanoma cell motility by modulating the miR‐192‐5p/EFEMP1 axis. Knockdown of EFEMP1 could reduce melanoma cell growth and invasiveness and promote apoptosis.^[^ ^134^ ^]^

In C32 melanoma cells, metformin decreases cell viability and DNA biosynthesis, while upregulating AMP‐activated protein kinase (AMPK) and proline dehydrogenase/proline oxidase (PRODH/POX) expression. This activation leads to PRODH/POX‐dependent ROS generation, initiating melanoma cell apoptosis.^[^ ^135^ ^]^ Moreover, metformin inhibits the phosphorylation of Smad3 and disrupts its interaction with lysine acetyltransferase 5 (KAT5), thereby attenuating KAT5‐mediated K333 acetylation of Smad3. This suppresses Smad3 transcriptional activity and TRIB3 expression, ultimately inducing autophagy and limiting melanoma progression.^[^ ^136^ ^]^ Metformin also modulates immune responses. For instance, metformin increases infiltration and proliferation of CD4^+^ and CD8^+^ T cells, reduces exhausted T cell populations, and facilitates the transition from M2‐tumor‐associated macrophages (M2‐TAMs) to M1‐TAMs, thereby improving the TME.^[^ ^137^ ^]^ Furthermore, metformin enhances the infiltration and activity of natural killer (NK) cells and T cells, improving immune surveillance in mouse models of lymphoma and metastatic melanoma.^[^ ^138^ ^]^ By downregulating EMT‐related factors and reducing the expression of N‐cadherin, vimentin, ZEB1, and ZEB2 at the metastasis sites, metformin furthermore inhibits melanoma cell metastasis.^[^ ^139^ ^]^

Collectively, these findings offer novel insights into the therapeutic potential of metformin in melanoma. However, pending definitive evidence of metformin's tumor‐specific survival benefits and a better understanding of its pharmacokinetic and mechanistic profiles in oncology, metformin may best be regarded as a promising adjuvant therapy for cancer patients with existing clinical indications for its use, owing to its established safety profile and potential to indirectly improve OS.

### 3.5 Cholesterol Modulators

The role of cholesterol in cancer development and the potential to therapeutically target cholesterol homeostasis remain contentious topics in cancer research. Large randomized controlled clinical trials investigating lovastatin for the prevention of cardiovascular disease have reported statistically significant reductions in melanoma incidence among patients receiving these medications.^[^ ^140^ ^]^ Recent literature indicates that statin use may improve outcomes in melanoma patients. A retrospective cohort analysis revealed that melanoma patients who used statins had significantly better 5‐year survival rates than non‐users.^[^ ^141^ ^]^ Additional studies have demonstrated that statin use is not only associated with favorable survival outcomes but also with a reduced risk of melanoma recurrence and metastasis. For instance, a prospective study found that the initiation of statin therapy prior to a melanoma diagnosis may decrease the risk of recurrence, particularly in men and in patients with ulcerated tumors.^[^ ^142^ ^]^ Furthermore, both univariate and multivariate analyses demonstrated that patients on statins were less likely to present with metastasis at the time of melanoma diagnosis.^[^ ^143^ ^]^ However, the association between statin use and melanoma risk remains controversial. A Mendelian randomization study revealed pharmacologically distinct effects: while HMGCR inhibitors (i.e., statins) potentially increase melanoma risk, proprotein convertase subtilisin/kexin type 9 (PCSK9) inhibitors (e.g., evolocumab, alirocumab) may confer protective effects.^[^ ^144^ ^]^ Clinical evidence appears similarly inconsistent. A retrospective analysis found no significant correlation between statin use and either sentinel lymph node (SLN) metastasis or recurrence rates in melanoma patients.^[^ ^145^ ^]^ Meta‐analyses have yielded conflicting results. One meta‐analysis of 11 studies found an elevated risk of melanoma with statin use,^[^ ^146^ ^]^ whereas another reported no statistically significant association (OR = 0.87, 95% CI: 0.61–1.23). These discrepancies may be due to methodological variations, including differences in study design, population characteristics, or statin dosage/duration assessments.

Extensive research has explored the mechanisms by which statins enhance the prognosis of melanoma patients. Through multiple pathways, statins can prevent the proliferation and dissemination of melanoma cells. For example, simvastatin has been shown to inhibit PARP1 expression, thereby inhibiting cell proliferation.^[^ ^147^ ^]^ Additionally, simvastatin activates a stress response cascade that promotes the synthesis of 15‐deoxy‐Δ[12,14]‐prostaglandin J_2_ (15d‐PGJ_2_) via p38 and COX‐2‐dependent pathways. Notable concentrations of 15d‐PGJ_2_ have been detected in the culture medium of melanoma cells, sufficient to activate caspase‐8 and the mitochondrial apoptosis pathway.^[^ ^148^ ^]^ Simvastatin also significantly downregulated NonO gene expression, a vital growth factor involved in splicing regulation.^[^ ^149^ ^]^ These results may account for the reduced proliferation of melanoma cells, suggesting a potential mechanism of action. Moreover, treatment of two human melanoma cell lines with statins resulted in a modest but statistically significant increase in the membrane expression of MHC class I chain‐related protein A (MICA). This upregulation enhances the susceptibility of melanoma cells to NK cell‐mediated lysis.^[^ ^150^ ^]^ Combination therapy with statins and other antitumor agents shows considerable promise. Co‐treatment with dacarbazine and statins significantly inhibited tumor growth and metastasis by suppressing the RhoA/RhoC/LIM domain kinase/serum response factor/c‐Fos signaling pathway, while enhancing the expression of p53, p21, p27, cleaved caspase‐3, and cleaved PARP1 in vivo. This combinatory approach also markedly improved survival in mice bearing metastatic tumors.^[^ ^151^ ^]^ In melanoma patients, maintaining stable blood cholesterol levels is important; however, controlling cholesterol levels within the TME is even more critical, as both excessively high and low cholesterol levels can impair the cytotoxic arm of antitumor immunity. Cholesterol‐enriched tumor tissues and elevated cholesterol within tumor‐infiltrating CD8⁺ T cells are positively correlated with increased expression of immune checkpoint molecules such as PD‐1, 2B4, TIM‐3, and LAG‐3. Adoptively transferred CD8^+^ T cells absorbed cholesterol, expressed high levels of these checkpoints, and became functionally exhausted upon entering the tumor environment.^[^ ^152^ ^]^ However, low cholesterol levels inhibit T cell proliferation and induce autophagy‐mediated apoptosis. In the TME, oxysterols mediate reciprocal alterations in the Liver X Receptor (LXR) and Sterol Regulatory Element‐Binding Protein 2 (SREBP2) pathways, causing cholesterol deficiency in T cells. This deficiency disrupts cellular metabolism and signaling pathways, driving T cell exhaustion and dysfunction.^[^ ^153^ ^]^ Low‐Density Lipoprotein Receptor (LDLR), the key transporter for cholesterol, plays a pivotal role in regulating CD8^+^ T cell antitumor activity. In addition to mediating cholesterol uptake, LDLR also interacts with the T cell receptor (TCR) complex, influencing TCR recycling and signaling, thus facilitating cytotoxic T lymphocyte (CTL) effector functions. However, the TME downregulates LDLR expression and TCR signaling in CD8⁺ T cells through tumor‐derived PCSK9, which binds LDLR and prevents its recycling to the plasma membrane. This process ultimately inhibits CTL function.^[^ ^154^ ^]^ Currently available lipid‐lowering agents, such as alirocumab and evolocumab, are monoclonal antibodies that target PCSK9, thereby preventing its interaction with LDLR. This, in turn, increases LDLR expression and promotes the uptake of low‐density lipoprotein cholesterol (LDL‐C).^[^ ^155^, ^156^ ^]^

Despite the clinical benefits of statins, the association between statin use and melanoma risk remains controversial and may be influenced by methodological variations. One study demonstrated that statins increase Foxp3 mRNA transcription and protein production, leading to an increased proportion of Tregs, which may contribute to immune suppression and potentially facilitate melanoma progression.^[^ ^157^ ^]^ Although both statins and PCSK9 inhibitors are lipid‐lowering agents, their distinct mechanisms of action warrant further investigation.

### 3.6 Antibiotics

Recent studies have highlighted the growing interest in the interaction between the gut microbiome and the immune system. Factors that disrupt this interaction, such as antibiotics to manage infections, may influence the immune response via the gut microbiota and interfere with tumor responses to ICIs. Exposure to antibiotics within 60 days prior to ICI initiation appears to be associated with poorer PFS and OS in melanoma patients receiving first‐line anti‐PD‐1 therapy. By contrast, antibiotics administered after the initiation of ICI therapy do not seem to impact PFS or OS.^[^ ^158^ ^]^ Antibiotic exposure was also associated with a greater incidence of moderate to severe immune‐mediated colitis.^[^ ^159^ ^]^ Moreover, individuals who received antibiotics had a significantly shorter time to discontinuation (TTD) of the ICI therapy, compared to those who did not.^[^ ^160^ ^]^ This population also exhibited higher rates of primary resistance and lower objective response rates.^[^ ^161^, ^162^ ^]^ In addition to compromising ICI efficacy, antibiotic use independently reduces the effectiveness of targeted therapies, particularly small‐molecule tyrosine kinase inhibitors (TKIs), leading to reduced PFS and OS in treated patients.^[^ ^163^ ^]^

Mechanistically, ICIs induce the remodeling of lymph nodes and the activation of dendritic cells (DCs), which facilitates the translocation of a selective subset of gut bacteria to extraintestinal tissues, thereby promoting optimal antitumor T cell responses in both the tumor‐draining lymph nodes (TDLNs) and primary tumor sites. Conversely, antibiotic treatment reduces the translocation of gut microbiota to mesenteric lymph nodes (MLNs) and TDLNs, resulting in diminished DC and effector CD8^+^ T cell responses, as well as attenuated sensitivity to immunotherapy.^[^ ^164^ ^]^ Moreover, antibiotic‐induced dysbiosis promotes distal tumor progression by modifying host cytokine levels, which subsequently reduces the expression of tumor endothelial adhesion molecules and impairs the activation of effector CD8^+^ T cells within the TME.^[^ ^165^ ^]^ Interestingly, while the antibiotic‐mediated depletion or gnotobiotic exclusion of fungi enhances antitumor immune responses to radiation therapy, the depletion of bacteria by antibiotics reduces treatment sensitivity and is correlated with an overgrowth of commensal fungi.^[^ ^166^ ^]^ Researchers have shown that microbioecological preparation may hold therapeutic potential in controlling tumor growth. For instance, astragalus polysaccharides, the main active constituents of *Astragalus membranaceus*, attenuate the immunosuppressive activity of myeloid‐derived suppressor cells (MDSCs) in melanoma‐bearing mice by remodeling the gut microbiota and altering fecal metabolites.^[^ ^167^ ^]^ Diosgenin, a natural steroidal saponin, exerts antitumor effects by modulating immune function and enhancing intestinal microbiota composition. Furthermore, the combination of PD‐1 antibody and diosgenin significantly exacerbated tumor necrosis and apoptosis by augmenting T cell‐mediated immune responses.^[^ ^168^ ^]^

The reviewed medications predominantly influence melanoma progression by remodeling immune microenvironment and inducing metabolic reprogramming, with some also exerting effects via epigenetic and transcriptional regulation (Table S2, Supporting Information). Although GCs are commonly used to mitigate irAEs associated with immunotherapy, high‐dose administration has been associated with reduced survival rates. This detrimental effect is attributed to the suppression of CD8^+^ TIL activity and activation of the ROCK1/2‐PI3K/Akt signaling pathway, which facilitates tumor metastasis. Therefore, optimizing GC dosing (e.g., <60 mg prednisone equivalent daily) may improve patient outcomes, while JAKi (e.g., tofacitinib) show promise for managing irAEs and enhancing immunotherapy efficacy by modulating immune cell function. However, further clinical validation is warranted. Antihistamines, by antagonizing the HRH1, inhibit macrophage polarization toward the immunosuppressive M2 phenotype, whereas HRH4 agonists have demonstrated promising antitumor potential. NSAIDs, through COX‐1/2 inhibition, reduce PGE2 production, thereby suppressing platelet aggregation and pre‐metastatic niche formation. Notably, NSAIDs have shown greater therapeutic benefits in female patients, underscoring the need for sex‐specific treatment strategies. Metformin induces apoptosis via modulation of miRNA expression and the AMPK/PRODH/POX pathway, while also enhancing T‐cell infiltration and promoting M1‐TAM polarization, thereby improving the immune microenvironment. Statins augment NK cell cytotoxicity by inhibiting the RhoA pathway and upregulating MICA; however, they also increase the proportion of Tregs through FOXP3 expression. More importantly, cholesterol levels must be carefully balanced: excessive levels can induce T‐cell exhaustion, whereas insufficient levels impair lymphocyte proliferation. Future research may focus on developing techniques for detecting and modulating localized cholesterol concentrations, although current efforts are primarily centered on validating and utilizing the therapeutic use of lipid‐lowering agents in melanoma management. Antibiotics disrupt gut microbiota composition, impairing DC and CD8^+^ T‐cell responses, thereby significantly diminishing the efficacy of ICIs and targeted therapies. Conversely, microbiota‐modulating agents such as astragalus polysaccharides may counteract immunosuppression and restore therapeutic responsiveness. Among these medications, we consider NSAIDs and cholesterol‐modulating drugs to be the most promising for future research. Clinical studies have demonstrated significant sex‐specific variations in the effects of NSAIDs on melanoma outcomes, yet the underlying mechanisms remain unexplored in basic research. Similarly, the association between statin use and melanoma risk remains controversial, possibly due to methodological heterogeneity across studies. These discrepancies may also reflect the mechanistic differences in how statin affects melanoma progression, warranting future investigation.

## 4 Metabolic Conditions

Obesity, dietary patterns, and physical activity are interrelated lifestyle factors that converge on the regulation of systemic metabolism, collectively shaping the risk, progression, and treatment response of melanoma. These factors modulate nutrient availability, energy balance, and immune function through both direct metabolic effects and indirect signaling pathways. Obesity, characterized by chronic low‐grade inflammation and adipokine imbalance, promotes melanoma progression by driving metabolic reprogramming (e.g., FAO) and activating pathways such as PI3K/Akt signaling, promoting tumor aggressiveness, and modulating immune responses. Diets affect metabolic homeostasis by reshaping gut microbiota, influencing insulin sensitivity, and altering nutrient availability, thereby impacting melanoma development and therapy response. Physical activity counteracts obesity‐related metabolic dysfunction by enhancing insulin sensitivity, reducing adiposity, and inducing metabolic competition that restricts tumor nutrient supply. Together, these factors underscore metabolism as a central hub linking obesity, diet, and exercise to melanoma pathogenesis, with hormones, adipocytes, and immune cell function systematically serving as key mediators.

### 4.1 Obesity

In recent decades, obesity has evolved into a chronic condition of growing global prevalence, significantly contributing to public health burdens across many nations.^[^ ^169^ ^]^ The internationally recognized metric for assessing obesity is the BMI, with overweight defined as a BMI between 25 and 30 kg m^−2^, and obesity as a BMI greater than 30 kg m^−2^. Both conditions are strongly linked to a broad spectrum of chronic and metabolic disorders. Moreover, obesity itself represents a chronic inflammatory state and is closely associated not only with an increased cancer risk but also with the accelerated progression of several malignancies,^[^ ^170^, ^171^ ^]^ including melanoma.^[^ ^172^ ^]^

Multiple studies have found that obesity correlates with an increased incidence of melanoma, with males appearing more susceptible to this association. In four Class A studies conducted between 2008 and 2013 involving patients from Europe, Canada, Australia, and the United States, BMI was found to be significantly associated with both melanoma incidence and mortality. More detailed stratification revealed that the elevated risk conferred by obesity was present only in men and not in women.^[^ ^173^ ^]^ Specifically, Nagel et al. reported that a BMI >23.8 in men was associated with a higher incidence of melanoma, whereas no significant relationship was observed in women.^[^ ^174^ ^]^ A meta‐analysis showed that the pooled effect sizes for overweight and obesity in men were 1.31 (95% CI: 1.18–1.45) and 1.31 (95% CI: 1.19–1.44), respectively. In contrast, no correlation was observed for women.^[^ ^175^ ^]^ However, these associations appear to have diminished in recent decades. A large prospective study involving over 57,000 participants demonstrated that obesity was not a significant risk factor for melanoma.^[^ ^176^ ^]^ Moreover, sensitivity analyses using two alternative Mendelian randomization (MR) approaches yielded similar results.^[^ ^177^ ^]^ Furthermore, a 40‐year Danish cohort study revealed that while obesity was positively associated with the risk of certain malignancies, but it was negatively associated with melanoma.^[^ ^170^ ^]^ We propose that the impact of obesity is multifactorial, involving not only physiological alterations but also lifestyle‐related factors. For example, individuals with obesity may engage in less outdoor physical activity, leading to reduced exposure to UVR,^[^ ^176^ ^]^ which may also partially explain the lack of association observed in women.^[^ ^175^ ^]^

The role of obesity in melanoma progression, prognosis, and response to ICIs remains controversial. While some studies have reported poorer outcomes in obese patients, others have demonstrated neutral or even beneficial effects. Elevated BMI has been associated with shorter OS in melanoma patients,^[^ ^178^ ^]^ thicker Breslow lesions,^[^ ^179^ ^]^ and increased risk of sentinel lymph node metastases.^[^ ^180^ ^]^ However, these associations often become non‐significant after adjusting for confounding factors such as chronic inflammation, as reflected by elevated C‐reactive protein (CRP) levels. This would be associated with decreased melanoma patient survival through chronic inflammation.^[^ ^178^ ^]^ Notably, skeletal muscle loss, rather than BMI alone, appears to be a stronger prognostic marker.^[^ ^181^ ^]^ Surprisingly, obesity does not appear to negatively affect ICI efficacy. Multiple studies have found no significant association between BMI and treatment outcomes in patients with metastatic melanoma receiving immunotherapy.^[^ ^182^, ^183^ ^]^ The concept of the “obesity paradox”, which denotes the paradoxical inverse relationship between obesity and mortality, is widely acknowledged in the cardiometabolic literature but is less frequently discussed in oncology.^[^ ^184^, ^185^ ^]^ In fact, overweight or obese patients may derive survival benefits from combination immunotherapy.^[^ ^186^ ^]^ Although studies on the impact of BMI on targeted therapy are sparse, existing data appear to mirror those seen in immunotherapy, indicating that elevated BMI does not necessarily result in worse outcomes.^[^ ^187^, ^188^ ^]^

Despite suggestive findings regarding the application of BMI as a predictive marker for melanoma patients' survival, particularly in terms of PFS and OS, its utility remains questionable due to inconsistent conclusions across studies. These inconsistencies are often attributed to methodological limitations such as reverse causation, selection bias, confounding variables, and the reliance on BMI as a surrogate for adiposity in cancer patients.^[^ ^189^ ^]^ For instance, male melanoma patients have been shown to benefit from higher BMI values (25–35 kg m^−2^), whereas no such benefit has been observed in female patients.^[^ ^190^ ^]^ This sex‐specific phenomenon may be partly explained by interindividual variability in fat distribution among individuals with the same BMI.^[^ ^191^ ^]^ Therefore, alternative anthropometric measurements, including body roundness index (BRI),^[^ ^192^ ^]^ skeletal muscle index (SMI),^[^ ^193^ ^]^ skeletal muscle density (SMD),^[^ ^194^ ^]^ and visceral fat index (VFI),^[^ ^195^ ^]^ may offer more accurate assessments of obesity and body composition. Notably, indicators of skeletal muscle mass (e.g., SMI and SMD) have been associated with improved prognosis in melanoma patients,^[^ ^194^, ^196^, ^197^ ^]^ and BRI has been proposed as a non‐invasive screening tool for estimating mortality risk,^[^ ^198^ ^]^ although further validation in independent melanoma cohorts has yet to be conducted. In summary, the impact of obesity on the efficacy of immunotherapy and the prognosis of melanoma remains inconclusive, due in part to the limitations of BMI as a measurement tool. As such, researchers investigating the relationship between body fat composition and melanoma outcomes should carefully select appropriate anthropometric indices.

**Figure 4:**
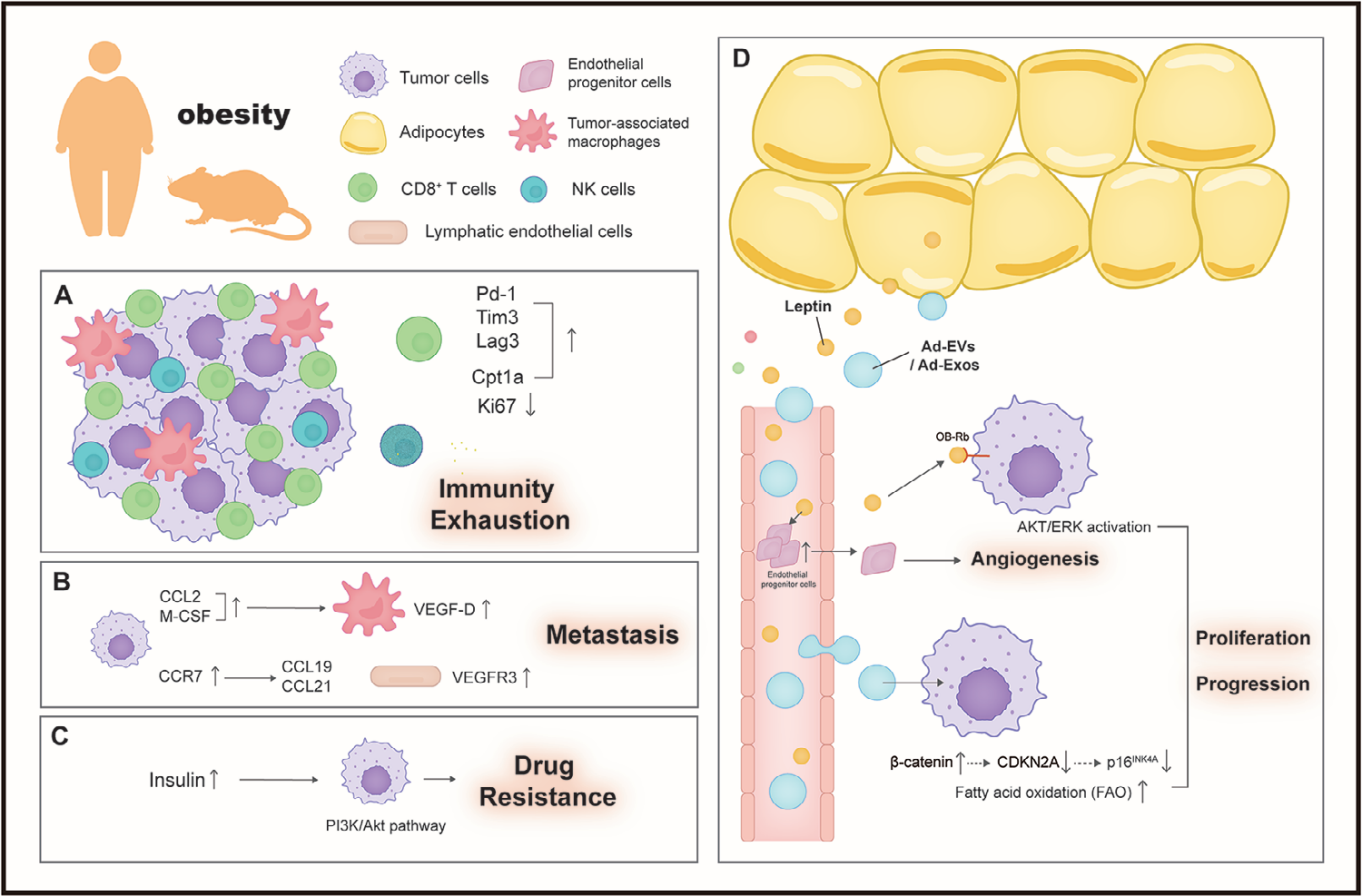
The mechanisms by which adipocytes contribute to melanoma progression through immune exhaustion, angiogenesis, lymphangiogenesis, and metastatic potential. A) Immunity exhaustion: CD8^+^ T cells exhibit exhaustion characterized by elevated PD‐1, Tim3, and Lag3 expression but reduced proliferation (Ki67). Additionally, increased Cpt1a expression drives FAO and exacerbates T cell exhaustion, impairing anti‐tumor immunity. B) Metastasis in TME: Adipocytes enhance the expression of CCL2, M‐CSF, and CCR7 mRNAs in melanoma cells, increasing the recruitment of M2‐TAMs. Adipocytes increase VEGF‐D expression in M2‐TAMs, enhancing angiogenesis and lymphangiogenesis. The CCL19/CCL21‐CCR7 axis and VEGFR3 expression in LECs promote lymph node metastasis. C) Hormonal change: excessive insulin results in drug resistance through PI3K/Akt pathway. D) Angiogenesis and lymphangiogenesis: Leptin, an adipokine secreted by adipocytes, stimulates melanoma growth by increasing circulating EPCs, promoting angiogenesis through vasculogenesis. Leptin interacts with its receptor (OB‐Rb) on melanoma cells, activating Akt and ERK pathways to enhance proliferation, drug resistance, and survival. Ad‐exos enriched in β‐catenin and FAO proteins induce metabolic reprogramming in melanoma cells, decreasing CDKN2A expression and p16^INK4A^ protein to increase motility. Obesity amplifies these processes via elevated ad‐exos and ad‐EVs, which also promote FAO‐dependent migration.

From a mechanistic perspective, the impact of obesity on melanoma largely stems from the cross‐talk between adipocytes and cancer cells, as well as alterations in the TME, which predominantly result in adverse effects (**Figure** **4**). Clinical studies have found an association between insulin resistance and cutaneous melanoma risk.^[^ ^199^ ^]^ Increased insulin levels dampen the therapeutic efficacy of dacarbazine and PLX4720 (mutant *BRAF* inhibitor) in melanoma cells via activation PI3K/Akt signaling pathway, suggesting that obesity‐associated hormonal changes might negatively affect melanoma therapy.^[^ ^200^ ^]^ Adipocytes, primarily located in adipose tissue, promote tumor progression through the secretion of chemokines,^[^ ^201^ ^]^ pro‐inflammatory cytokines,^[^ ^202^ ^]^ and adipocyte‐derived extracellular vesicles (Ad‐EVs).^[^ ^203^ ^]^ Researchers have found that mature adipocytes (MAs) enhance the expression of C‐C motif chemokine ligand 2 (CCL2), Macrophage colony‐stimulating factor (M‐CSF), and C–C motif chemokine receptor 7 (CCR7) mRNAs in melanoma cells, leading to an increase in M2‐TAMs. Furthermore, MAs upregulate vascular endothelial growth factor D (VEGF‐D) mRNA in M2‐TAMs, thereby promoting angiogenesis, lymphangiogenesis, and enhancing metastatic potential. They also stimulated the CCL19/CCL21–CCR7 axis and VEGF receptor (VEGFR) mRNA in LECs, contributing to increased lymph node metastasis.^[^ ^204^ ^]^ In the context of cytokines, obesity leads to dysregulated secretion of adipokines and inflammatory cytokines such as tumor necrosis factor‐alpha (TNF‐α) and IL‐6, which forster a pro‐TME.^[^ ^205^ ^]^ Leptin, an adipokine whose levels in proportional to adipose tissue mass and circulates in the blood, has been implicated in melanoma growth, although its presence is not essential for tumor initiation.^[^ ^206^ ^]^ High leptin and resistin (another adipokine) levels in obesity impair the efficacy of chemotherapeutic agents like dacarbazine in melanoma cells, increasing drug resistance and reducing treatment response.^[^ ^207^ ^]^ And the increased serum leptin levels were correlated with PD‐1 expression on CD8^+^ T cells in obese donors and diet‐induced obesity (DIO) mice, driving T cell exhaustion within TME.^[^ ^208^ ^]^ Moreover, researchers found that leptin has also been shown to promote vasculogenesis by increasing circulating endothelial progenitor cells (EPCs)^[^ ^209^ ^]^ and directly interacts with its long‐form receptor OB‐Rb on melanoma cell surfaces. Depletion of leptin in adipocytes or knockdown of OB‐Rb in melanoma cells using siRNA abrogated Akt and ERK activation, cell proliferation, and drug resistance.^[^ ^210^ ^]^ Ad‐EVs convey β‐catenin protein to melanoma cells, where it accumulates in the nucleus and suppresses CDKN2A expression, reducing p16^INK4A^ protein and enhancing cell motility. In obesity, these effects are amplified due to an increased quantity of Ad‐EVs.^[^ ^203^ ^]^ Exosomes, a subset of EVs with an average diameter of ≈100 nm,^[^ ^211^ ^]^ have emerged as critical mediators in cancer progression.^[^ ^212^ ^]^ Adipocyte‐derived exosomes (ad‐exos), which are enriched in proteins related to fatty acid oxidation (FAO), induce metabolic reprogramming of melanoma cells toward FAO, thereby promoting tumor aggressiveness. Notably, in both obese mice and humans, the quantity and FAO‐dependent effects of ad‐exos are markedly amplified.^[^ ^213^ ^]^ In another way, obesity also profoundly alters the number and function of immune cells within the TME. In DIO mice, CD8⁺ T cells exhibited features of immune exhaustion, including elevated expression of Pd‐1, T cell immunoglobulin and mucin domain‐3 (Tim3), and lymphocyte activation gene‐3 (Lag3), alongside reduced Ki‐67 expression, indicating impaired proliferation. Moreover, the expression of Cpt1a, a key gene in FAO, was elevated in the tumor tissues and CD8^+^ T cells of DIO mice.^[^ ^214^ ^]^ Cpt1a has been identified as upregulated in the early stages of T cell exhaustion and is considered a potential driver of metabolic reprogramming exhaustion with this phenotype.^[^ ^215^ ^]^ Inhibition of PPARα/δ or blockade of mitochondrial lipid transport restored NK cell cytotoxicity.^[^ ^216^ ^]^

There is a paradoxical relationship between obesity and response to ICIs, wherein obese patients occasionally exhibit improved outcomes compared to lean individuals.^[^ ^188^, ^196^ ^]^ A key mechanistic link involves leptin and T cell immunity. On one hand, leptin promotes memory T cell exhaustion by enhancing PD‐1 expression through the STAT3 signaling pathway, thereby impairing anti‐tumor immunity. On the other hand, this leptin‐driven upregulation of PD‐1 also renders T cells more reliant on the PD‐1 axis for immune regulation, paradoxically making them more responsive to PD‐1 blockade therapy.^[^ ^214^ ^]^ Clinical data show that serum leptin levels above the median are associated with a reduced risk of metastasis in patients with uveal melanoma, independent of sex and cancer stage, in a separate cohort (*n* = 80).^[^ ^217^ ^]^ Intriguingly, tumors harboring *BAP1* mutations and epithelioid histology exhibited higher leptin receptor mRNA expression was inversely correlated with serum leptin levels, suggesting a potential compensatory feedback mechanism between systemic and tumor‐intrinsic leptin signaling.

### 4.2 Diets and Nutrients

Dietary habits are the major factor influencing obesity. Different dietary patterns, especially healthy ones such as the Mediterranean diet (MD), vegetarian diet, Japanese diet, gut microbiota‐regulating diets, and the very‐low‐carbohydrate ketogenic diets, have been demonstrated to lower the risk of several cancers and reduce associated mortality.^[^ ^218^ ^]^ In recent years, the influence of dietary patterns and specific nutrients on melanoma has gained increasing attention. Given this, we focus on three common dietary patterns: caloric restriction, the Mediterranean diet, and high‐fat diets (HFD). We also summarize their effects on melanoma progression, as well as the underlying biological mechanisms involved.

#### 4.2.1 Caloric Restriction

Caloric restriction (CR) refers to a dietary regimen that reduces daily caloric intake by 20–40% while maintaining adequate nutritional intake. It is believed that the CR diet could extend lifespan and decrease the incidence of tumors and other chronic diseases in experimental models.^[^ ^219^ ^]^ In contrast, high caloric intake is positively associated with an increased risk of melanoma,^[^ ^220^ ^]^ consistent with the established link between obesity and malignancy. A CR diet has been shown to inhibit the growth and metastasis of melanoma by reducing the size and density of microvessels. Mechanistically, these effects may be mediated through suppression of the NF‐κB pathway and extracellular matrix remodeling, leading to the preserved E‐cadherin expression and reduced local invasion.^[^ ^221^ ^]^ However, the role of CR in modulating anti‐tumor immunity remains controversial. In patients experiencing sarcopenia or cachexia due to prolonged caloric deficiency, immune responses may be compromised and may have a poorer response to immunotherapy, with decreased immune cell infiltration in the TME and impaired T cell activation.^[^ ^222^, ^223^ ^]^ In melanoma, the efficacy of anti‐PD‐1 antibody therapy was diminished under CR conditions, accompanied by altered mitochondrial function in CD8⁺ T cells.^[^ ^224^ ^]^ Notably, PD‐1 deficiency has been linked to enhanced survival of memory CD8⁺ T cells through increased glycolysis and impaired fatty acid metabolism^[^ ^225^ ^]^; thus, the energy‐restricted state induced by CR may hinder CD8⁺ T cell surveillance and reduce the efficacy of immunotherapy. Further studies are warranted to clarify these mechanisms. CR also affects the gut microbiota, which may indirectly influence melanoma. Evidence suggests that CR can reshape the gut microbial community, notably increasing the abundance of *Lactobacillus* species (*p* < 0.05), thereby alleviating microbial dysbiosis associated with colorectal cancer.^[^ ^226^ ^]^ However, its impact on melanoma appears to be less direct. A recent study indicated that dietary factors—including the consumption of plant‐based foods, dairy, and fats—as well as gastrointestinal function, are significantly associated with the outcomes of ICI therapy. In particular, melanoma patients consuming low amounts of dairy (≤1 serving d^−1^) exhibited a twofold increase in response to anti‐PD‐1 therapy compared to those with higher dairy intake (2–3 servings d^−1^).^[^ ^227^ ^]^ Although low dairy intake may resemble a form of CR, a direct mechanistic link has not yet been established.

Intermittent fasting has recently been proposed as an alternative to long‐term CR diets. This approach involves fasting on specific days to reduce overall caloric intake,^[^ ^228^ ^]^ which has been shown to protect normal cells, both in mice and potentially in humans, against the adverse effects of various chemotherapeutic agents. For instance, a preclinical study indicated that cyclic fasting increased the susceptibility of melanomas in mice to the chemotherapeutic agent Adriamycin.^[^ ^229^ ^]^ In animal models, intermittent fasting also reduced the melanoma growth and enhanced susceptibility to the targeted medication sorafenib.^[^ ^230^ ^]^ Additionally, periodic fasting‐mimicking diet (FMD) cycles, either administered alone or in conjunction with anti‐OX40 or anti‐programmed death‐ligand 1 (PD‐L1) agents, have demonstrated significantly greater efficacy than ICIs alone in inhibiting melanoma growth in murine models. Notably, FMD treatment has also been shown to effectively prevent or reverse ICI‐induced cardiac fibrosis, necrosis, and hypertrophy.^[^ ^231^ ^]^ These findings underscore the potential of intermittent fasting, particularly when compared to a continuous CR diet, for improved immunotherapeutic outcomes in melanoma.

#### 4.2.2 Mediterranean Diet

Numerous studies have demonstrated the prophylactic effect of the Mediterranean diet against various neoplasms and chronic diseases. The dietary pattern is characterized by a high intake of vegetables, fruits, fish, grains, legumes, and olive oil; moderate consumption of dairy products; and low intake of red meat, desserts, desserts, sugar‐sweetened beverages, and refined grains. Researchers have suggested that several dietary components intrinsic to the MD—particularly the fish and shellfish rich in n‐3 fatty acids, with a high intake of vegetables such as carrots, cruciferous, and leafy greens—may offer protection against cutaneous melanoma.^[^ ^232^ ^]^ While earlier studies primarily focused on individual food items, Mahamat‐Saleh et al. employed a 9‐unit dietary score encompassing the intake of fruits, vegetables, legumes, cereal products, olive oil, fish, dairy products, meat products, and alcohol. Their findings revealed a negative correlation between adherence to the MD and the risk of developing melanoma.^[^ ^233^ ^]^ Moreover, a multicenter cohort study (the PRIMM study) also reported a positive association between the MD and improved response to ICI therapy in melanoma patients.^[^ ^234^ ^]^

The protective effects of the MD against melanoma are closely associated with gut microbiota modulation. Notably, the high content of vegetables in the MD provides substantial dietary fiber. A clinical study involving 128 melanoma patients receiving ICI therapy demonstrated that higher dietary fiber intake significantly correlates with improved PFS.^[^ ^235^ ^]^ This high‐fiber diet may offer protection against irAEs, such as colitis and diarrhea, particularly in patients treated with ipilimumab.^[^ ^236^ ^]^ Moreover, the MD has been linked to an increased abundance of gut bacteria that produce short‐chain fatty acids (SCFAs).^[^ ^237^ ^]^ Several studies have demonstrated that SCFA levels may serve as predictors of immunotherapy responses,^[^ ^235^, ^238^ ^]^ with higher concentrations of specific SCFAs associated with prolonged PFS in patients with various solid tumors treated with PD‐1 inhibitors.^[^ ^239^ ^]^ Conversely, other findings suggest that elevated systemic levels of SCFAs may be linked to resistance against Cytotoxic T‐lymphocyte‐associated protein 44 (CTLA‐4) blockade. In murine models, butyrate was shown to inhibit anti‐CTLA‐4‐induced upregulation of CD80/CD86 on DCs and ICOS (CD278) on T cells, as well as suppress the accumulation of tumor‐specific and memory T cells. In human patients, higher circulating butyrate levels moderate the ipilimumab‐induced accumulation of memory T cells (ICOS^+^ CD4^+^ T cells), and interleukin‐2 (IL‐2) production.^[^ ^240^ ^]^ Collectively, these findings suggest that while SCFAs may contribute to the beneficial immunomodulatory effects of the MD, they might also limit the efficacy of anti‐CTLA‐4 therapy, necessitating further investigation into the underlying mechanisms involved.

#### 4.2.3 High‐Fat Diet

Despite extensive research, evidence regarding the association between fatty acid consumption and melanoma remains conflicting. Experimental studies have suggested that metabolic stress induced by HFD promotes melanoma growth in the bone marrow through an increase in bone marrow adipocytes and IL‐6–JAK2–osteopontin‐mediated activation of tumor cells and osteoclast differentiation.^[^ ^241^ ^]^ According to a prospective study on an Australian population, high omega‐3 and omega‐6 fatty acid intakes may contribute to the presentation of thicker melanomas (>2mm).^[^ ^242^ ^]^ However, a MR study found no significant association between polyunsaturated fatty acid levels and melanoma incidence.^[^ ^243^ ^]^ HFD are widely considered a major contributor to obesity, which, as previously discussed, plays a significant role in melanoma development and progression. Therefore, researchers studying the relationship between HFD and melanoma should carefully control for the confounding effects of obesity in experimental designs, as precise, obesity‐independent studies in this context are still lacking.

When investigating the role of an HFD on the microbiota, it is essential to exclude the effects of obesity. The HFD has been shown to promote tumor progression in the small intestine of genetically susceptible *K‐ras* G12D mice, independent of obesity.^[^ ^244^ ^]^ In the context of melanoma, microbiota from leptin‐deficient (ob/ob) or adiponectin‐deficient (AdpKO) mice can stimulate melanoma development in lean wild‐type (WT) mice fed an HFD.^[^ ^245^ ^]^ Furthermore, the HFD facilitates the formation of a lung pre‐metastatic niche and metastasis. Glycyrrhizic acid (GA) has been shown to attenuate HFD‐enhanced recruitment of MDSCs, expression of the pro‐metastatic protein S100A8/A9, and overall metastatic burden in 4T1 breast cancer and B16F10 melanoma. This effect is accompanied by alterations in gut microbiota and polarization of colonic macrophages away from the M1‐like phenotype.^[^ ^246^ ^]^

Overall, dietary habits significantly influence melanoma risk and clinical outcomes. Well‐informed patients who adopt beneficial dietary changes and consider appropriate nutritional supplementation may actively contribute to their own treatment, potentially improving therapeutic efficacy. Nonetheless, further research is required to define dietary patterns that may reduce melanoma risk and enhance prognosis.

### 4.3 Exercise

A growing body of preclinical and clinical evidence indicates that physical activity (PA) exerts potent preventive effects, reducing the incidence and recurrence rates of many tumors.^[^ ^247^, ^248^ ^]^ Nevertheless, the population‐based studies specifically examining the association between exercise and melanoma remain limited, and the results are not uniformly consistent.

The role of exercise in melanoma is still under debate. A prospective observational study demonstrated that high levels of leisure‐time physical activity were associated with a reduced incidence of 13 different cancer types, but paradoxically linked to an increased risk of melanoma (HR = 1.27, 95% CI: 1.16‐1.40).^[^ ^249^ ^]^ This association may reflect increased UV exposure during outdoor exercise, underscoring the importance of sun protection practices.^[^ ^250^ ^]^ However, more recent evidence does not support this association and highlights the role of PA in both the prevention and progression of melanoma. A population‐based cohort study conducted in Norway demonstrated that overall PA was not associated with melanoma risk when considering all body sites collectively; however, a reduced risk was observed for melanomas located on the upper limbs (HR = 0.70 for high vs low PA). Additionally, overall PA is negatively correlated with the incidence of sunburn, while sunburn incidence is positively associated with behaviors such as sunbathing vacations and indoor tanning.^[^ ^251^ ^]^ These findings suggest that sunburn, rather than physical activity itself, may act as a confounding factor and does not negate the potential protective role of physical activity in melanoma prevention.

The impact of exercise on melanoma prognosis also remains a contentious issue. A population‐based study demonstrated that exercise pre‐diagnosis PA had no significant influence on melanoma‐specific mortality.^[^ ^252^ ^]^

While in animal experiments, wheel‐running distance has been found to have a negative association with cancer growth in older mice (aged 20 and 28 months), but no such correlation was observed in younger mice.^[^ ^253^ ^]^ In several rodent studies involving orthotopic breast^[^ ^254^ ^]^ and prostate tumors^[^ ^255^ ^]^ revealed that post‐implantation exercise may alter the TME, enhancing its susceptibility to therapy by increasing tumor perfusion and reducing hypoxia. However, in melanoma models, exercise did not significantly affect tumor hypoxia, perfusion, or growth rate.^[^ ^256^ ^]^ The role of exercise in immunotherapy is also not fully understood. For instance, the combination of exercise and anti‐PD‐1 therapy increased the percentage of CD8^+^ T cells in mice with EO771 breast tumor‐bearing mice, but not in mice with B16F10 melanoma.^[^ ^257^ ^]^ Conversely, another study demonstrated that exercise exerted evident anti‐tumor effects and favorable biosafety in the B16F10 homograft malignant melanoma model, primarily by alleviating hypoxia and enhancing tumor immune infiltration within the tumor.^[^ ^258^ ^]^

A substantial body of foundational research has provided additional evidence supporting the benefits of exercise for patients with melanoma. In the B16F10 homograft malignant melanoma model, low‐ to moderate‐intensity swimming led to downregulation of hypoxia‐related pathways, a notable increase in cytotoxic T cells, and a reduction in Tregs, indicating an improved tumor immune microenvironment.^[^ ^258^ ^]^ Furthermore, voluntary wheel running was shown to inhibit melanoma initiation and progression via direct regulation of NK cell trafficking. This was characterized by epinephrine‐dependent mobilization of NK cells into circulation and IL‐6‐dependent recruitment to tumor sites.^[^ ^259^ ^]^ In another study, exercise suppressed tumor growth and enhanced CD8⁺ T cell infiltration in YUMMER murine melanoma models.^[^ ^260^ ^]^ These exercise‐induced metabolic reprogramming has been proposed to increase systemic nutritional demands—such as catabolic activity, glucose uptake, mitochondrial function, and GLUT expression—thereby creating metabolic competition that restricts nutrient availability to tumors and forms a metabolic barrier around them.^[^ ^261^ ^]^

Recent genomic studies have investigated molecular differences between melanoma patients with and without regular exercise. These analyses revealed genes enriched in NF‐κB signaling, chemokine pathways, and immune responses,^[^ ^262^, ^263^ ^]^ offering potential exercise‐related biomarkers for prognosis prediction. The primary anticancer mechanism of PA is likely the maintenance of healthy body weight and reduction of adiposity.^[^ ^264^ ^]^ Additionally, exercise exerts significant metabolic effects, including enhanced insulin sensitivity and reduced plasma insulin level; modulation of insulin‐like growth factor (IGF) axis (decreased IGF‐1 and increased IGFBP‐3)^[^ ^265^ ^]^; and notably, suppression of sex hormone synthesis (e.g., estrone, estradiol, and testosterone).^[^ ^264^ ^]^ Given that estrogen has previously been suggested as a protective factor, further research is warranted to elucidate these hormonal mechanisms. It is important to note that variations in study design—particularly the classification of exercise intensity and duration—can lead to inconsistent conclusions. A more standardized and scientifically rigorous approach to defining exercise parameters is needed. Moreover, the effects of exercise on immunotherapy and targeted therapy remain insufficiently explored and merit further investigation.

Current research on the obesity–melanoma relationship is constrained by two fundamental issues. The first stems from the limitations of BMI as a defining metric, which fails to capture the heterogeneity of fat distribution and inadequately controls for confounding variables. For instance, while some studies report enhanced immunotherapy efficacy in males with BMI>35 kg/m^2^, this association is absent in females—a discrepancy potentially attributable to sex‐specific differences in adiposity, as women typically exhibit higher body fat percentages than men at equivalent BMI values. The second issue concerns obesity's dual‐pathway influence on tumor progression. At the cellular level, adipocytes contribute to an immunosuppressive tumor microenvironment through the secretion of adipokines (leptin), pro‐inflammatory cytokines (IL‐6, TNF‐α), and extracellular vesicles (Ad‐EVs), while simultaneously inducing metabolic quiescence in tumor cells via downregulation of oxidative phosphorylation and other key metabolic pathways.^[^ ^266^ ^]^ Systemically, obesity interacts with insulin resistance and lipid metabolism dysregulation, though conventional metrics like BMI poorly reflect this underlying metabolic complexity.

These limitations underscore the need for more precise metabolic indicators, such as visceral adiposity index (VAI) and SMI, as well as a more rigorous investigation of obesity's interactions with other metabolic regulators. As a central metabolic hub, obesity mediates the tumor‐modulating effects of both dietary patterns and physical activity through adipose tissue remodeling. HFD promote melanoma progression via expansion of bone marrow adipocyte and activation of IL‐6–JAK2–osteopontin axis, whereas exercise may restrict tumor nutrient availability by increasing systemic metabolic demand (Table S3, Supporting Information). Consequently, future research should prioritize elucidating the integrated regulatory mechanisms driving obesity‐associated metabolic reprogramming, rather than examining isolated factors in a reductionist manner.

## 5 Behavioral Risk Profiles

### 5.1 Smoking

Smoking has been conclusively demonstrated as a significant risk factor for various cancers, including lung, laryngeal, esophageal, liver, pancreatic, and gastric malignancies.^[^ ^267^ ^]^ However, the relationship between smoking and melanoma incidence remains inconsistent across studies. While some investigations suggest a positive association, others report an inverse correlation. More recent analyses tend to support the latter. A case‐control study of 1157 melanoma patients and 5595 controls reported a strong inverse association between cigarette smoking and melanoma risk in men, with no clear association in women.^[^ ^268^ ^]^ Another large case‐control study showed that the current and former smokers had an OR of 0.5 (95% CI: 0.39–0.64) for melanoma compared to nonsmokers.^[^ ^269^ ^]^ In line with this, several prospective studies also indicated a reduced risk of melanoma among smokers.^[^ ^270^ ^]^ Additionally, some evidence suggests that a longer duration and number of cigarettes smoked may be associated with a further decrease in melanoma incidence.^[^ ^271^ ^]^

Multiple studies have demonstrated an inverse correlation between smoking and melanoma incidence—a counterintuitive finding that challenges conventional understanding. To explain this, several hypotheses have been proposed. Many studies may have failed to adequately account for confounding factors such as gender, UV exposure history, skin phototype, or the frequency of blistering sunburns, which could bias the observed relationship between cigarette smoking and melanoma. Nevertheless, after adjusting for age, sex, race, skin type, UV exposure, and sunburn history, results remained consistent with earlier research.^[^ ^272^ ^]^ Moreover, MR analyses revealed that high‐frequency smoking is significantly associated with a reduced risk of melanoma incidence (OR = 0.67, 95% CI: 0.51–0.89).^[^ ^273^ ^]^ Interestingly, although men constitute the majority of smokers, a prospective observational study in women also reached the same conclusion.^[^ ^274^ ^]^ Although limited in number, studies that rigorously control for confounding variables have independently validated the protective association between smoking and melanoma risk.

When examining the effect of smoking on melanoma progression, the evidence consistently suggests that smoking accelerates tumor development. Smoking has been confirmed as a key predictor of both melanoma‐specific and all‐cause mortality. Smokers tend to present with significantly thicker tumors and have a higher risk of death compared to non‐smokers.^[^ ^275^ ^]^ Additionally, smokers more frequently exhibit ulcerated lesions and are more likely to have lymph node metastases, further supporting the role of smoking in promoting melanoma progression.^[^ ^276^ ^]^

However, the effect of smoking on patient prognosis remains controversial. One prospective study observed a 23% lower mortality rate among smokers than non‐smokers, although the absence of a clear dose–response relationship weakened the evidence for causality.^[^ ^270^ ^]^ In contrast, several other studies have identified smoking as an important factor contributing to the increased mortality in melanoma patients. For example, smoking at the time of diagnosis was independently associated with increased melanoma‐specific mortality.^[^ ^275^ ^]^ The hazard ratios of death from melanoma in persistent and former smokers were 1.81 and 1.75 times, respectively, compared to non‐smokers, suggesting that smoking is an independent modifiable poor prognostic factor in cutaneous melanoma.^[^ ^277^ ^]^ Conversely, some researchers have reported no significant association between smoking and melanoma mortality. For instance, Gibson et al. found that smoking increased all‐cause mortality but had no effect on melanoma‐specific mortality in a population‐based case‐control study.^[^ ^269^ ^]^ Similarly, in another cohort study comparing 114 pairs of current smokers vs. never smokers, 113 pairs of current smokers vs former smokers, and 174 never smokers vs former smokers, smoking status was not associated with SLN metastasis, disease‐free survival (DFS), melanoma‐specific survival, or OS in any group.^[^ ^278^ ^]^ Despite these inconsistencies, smoking is clearly linked to increased all‐cause mortality and premature death.^[^ ^279^ ^]^ Therefore, clinicians should routinely inquire about smoking habits and provide cessation support.

The mechanism by which smoking may reduce the incidence of melanoma remains incompletely understood, and several hypotheses have been proposed to explain this phenomenon from various perspectives. One possible explanation is the immunosuppressive effect of smoking, which may hypothetically protect melanocytes from UVR‐induced inflammatory responses.^[^ ^271^, ^280^ ^]^ Tobacco also contains significantly higher levels of lithium than most other plants. Inhaled lithium may account for the reduced melanoma risk in smokers by inhibiting glycogen synthase kinase‐3 beta (GSK‐3β), thereby enhancing β‐catenin activity.^[^ ^281^ ^]^ Activation of the Wnt/β‐catenin pathway is associated with the upregulation of several genes that are typically absent in aggressive melanomas compared to normal melanocytes.^[^ ^282^ ^]^ In lithium‐treated tumor xenografts, the levels of autophagy markers LC3β and LAMP1 were significantly elevated compared to controls. Lithium treatment also increased caspase‐3 expression and promoted apoptotic cell death in tumor cells. Consequently, lithium carbonate may be a compound capable of inhibiting melanoma cell proliferation and promoting cell death through the induction of autophagy and apoptosis.^[^ ^283^ ^]^ Furthermore, lithium significantly enhanced RSL3‐induced ferroptosis in vitro, as evidenced by increased mitochondrial peroxide levels, lipid peroxidation, and mitochondrial abnormalities. Additionally, lithium inhibited the proliferation and migration of B16F10 melanoma cells in a dose‐dependent manner. Mechanistically, it modulated intracellular ferrous ion levels by downregulating ferritin heavy chain (Fth1), a key regulator of iron homeostasis. The combination of lithium and RSL3 significantly suppressed tumor growth in vivo, which was associated with reduced Fth1 expression and increased iron deposition in the spleen and liver, thereby highlighting a novel interaction between lithium and iron metabolism. Notably, this combination also enhanced CD8^+^ T cell infiltration and IFN‐γ expression in the TME, particularly among cytotoxic effector CD8^+^ T cells.^[^ ^284^ ^]^

In addition to lithium, other tobacco‐derived compounds such as flavonoids—particularly rutin and quercetin—have been shown to exert anti‐melanoma effects. These flavonoids inhibit the melanoma cell growth via various mechanisms. Preliminary in vitro studies indicate that rutin reduces the viability of the RPMI‐7951 and SK‐MEL‐28 melanoma cell lines, while also inducing apoptosis and cellular senescence, suggesting anti‐melanoma properties.^[^ ^285^ ^]^ Quercetin exerts broad inhibitory effects on melanoma cells, including reduced proliferation, induction of apoptosis, and diminished migratory and invasive capability. Mechanistically, quercetin inhibits the STAT3 signaling pathway by interfering with STAT3 phosphorylation and nuclear translocation. This downregulates the transcriptional activity of STAT3 and its downstream targets, such as Mcl‐1 (involved in cell survival), and MMP‐2, MMP‐9, and VEGF, which regulate migration and invasion.^[^ ^286^ ^]^

From an epidemiological perspective, smoking increases overall mortality, which may introduce a survival bias that partially obscures the association between smoking and melanoma‐related mortality.^[^ ^277^ ^]^ The mechanism by which smoking promotes melanoma metastasis has been elucidated in part by Nguyen et al., who found that nicotine upregulates the activity of α9 nicotinic acetylcholine receptor (α9‐nAChR). This receptor activation triggers the Akt and ERK signaling pathways, promotes PD‐L1 expression via STAT3 activation, and induces EMT‐induced melanoma migration.^[^ ^287^ ^]^ In summary, the inverse correlation between smoking and melanoma incidence is striking, yet its prognostic significance remains to be fully elucidated. Moreover, the impact of smoking on the efficacy of immunotherapy and targeted therapies warrants further investigation.

### 5.2 Alcohol Consumption

In 1988, the International Agency for Research on Cancer (IARC) classified alcohol and its primary metabolite acetaldehyde (AcAH), as Group 1 carcinogens. Approximately 4% of newly developed cancers are attributable to alcohol consumption, particularly tumors of the gastrointestinal tract.^[^ ^288^ ^]^ Numerous studies have identified that alcohol consumption is a risk factor for melanoma. An MR study reported a causal relationship between alcohol intake and melanoma incidence, which in turn, indicating a 2.23‐fold increased risk of developing melanoma.^[^ ^289^ ^]^ In line with this, researchers demonstrated that, compared to nondrinkers, the pooled multivariate‐adjusted HRs for individuals consuming ≥20 g/day of alcohol were 1.02 for melanomas of the head, neck, and extremities, and 1.73 for melanomas of the trunk.^[^ ^290^ ^]^ As expected, the incidence of melanoma rises with increasing alcohol consumption. A prospective cohort study initiated in 1992 across ten European countries also revealed a positive relationship between alcohol consumption and the risk of skin cancer.^[^ ^291^ ^]^ Increased current and lifetime alcohol consumption, as well as a preference for white wine or liquor, were linked to a heightened risk of both malignant melanoma and non‐melanoma skin cancer.^[^ ^292^ ^]^ Several meta‐analyses have further confirmed the positive association between alcohol consumption and melanoma development, with the amount of alcohol consumed emerging as a significant risk factor.^[^ ^293^, ^294^, ^295^ ^]^ Nevertheless, the possibility of residual confounding and bias cannot be entirely excluded. Further research is warranted to validate these findings, clarify the role of different types of alcoholic beverages, and explore their interactions with established melanoma risk factors.

Alcohol consumption is generally not recommended for melanoma patients due to its potential adverse effects on disease development and prognosis. However, prospective cohort studies specifically examining the association between alcohol intake and melanoma outcomes remain limited. Animal studies have demonstrated that alcohol may contribute to cancer progression through multiple mechanisms, particularly by modulating the immune function. Study has showed that chronic alcohol consumption inhibits the proliferation of memory T cells, accelerates the decline of IFN‐ γ‐producing CD8^+^ T cells, and increases the population of MDSCs, all of which may facilitate melanoma progression and reduce survival.^[^ ^296^ ^]^ In melanoma‐bearing mice exposed to alcohol, the expression of sphingosine‐1‐phosphate receptor 1 (S1PR1) and sphingosine‐1‐phosphate lyase 1 (SGPL1) in splenocytes was downregulated, thereby impairing B cell migration from the spleen to peripheral circulation and resulting in reduced peripheral B cell counts.^[^ ^297^ ^]^ Chronic alcohol exposure also decreased the percentage and number of CD62L^+^NK cells in the spleen, which are crucial for NK cell trafficking into lymph nodes.^[^ ^298^ ^]^ In addition to its effects on immune cells, Ethanol has been found to increase the expression of VEGF and Fms‐like tyrosine kinase 1 (FLT1), which may directly promote tumor growth and angiogenesis. In animal experiments, ethanol exposure led to a 2.16‐fold increase in tumor weight compared to controls, accompanied by significantly elevated levels of VEGF and FLT1 expression in melanoma tissues.^[^ ^299^ ^]^ In vitro studies using FEMX‐I cells further demonstrated that ethanol induces CD271 expression through NF‐κB activation, suggesting a possible molecular link between ethanol exposure and melanoma progression.^[^ ^300^ ^]^ Moreover, AcAH, the primary metabolite of ethanol, is a plausible carcinogen, which has been implicated in DNA damage, inhibition of DNA synthesis, and disruption of DNA repair mechanisms repair.^[^ ^301^ ^]^ Notably, similar to the liver, the skin expresses enzymes responsible for the metabolism of ethanol and AcAH. These enzymes play a key role in detoxification and prevention of AcAH accumulation.^[^ ^302^ ^]^ However, the expression of aldehyde dehydrogenase 2 (ALDH2), a critical enzyme in AcAH detoxification, is reduced in melanoma tissues, potentially compromising this protective mechanism.^[^ ^303^ ^]^

Studies investigating alcohol consumption often rely on questionnaire‐based and self‐reported data, which may introduce recall bias and inconsistencies, particularly regarding the type and quantity of alcohol consumed. These limitations highlight the need for more rigorous, mechanistic studies to elucidate the effects of ethanol and its metabolites on melanoma development and progression. Based on the current body of evidence, it is advisable for patients with melanoma to minimize or abstain from alcohol consumption

### 5.3 Coffee Consumption

Compared to alcohol, coffee is a more favorable beverage due to its well‐documented health benefits, including inverse associations with the risk of diabetes, Parkinson's disease, and various cancers. Coffee contains numerous phytochemicals, such as caffeine, chlorogenic acid, and caffeic acid,^[^ ^304^ ^]^ that exhibit potential anticancer properties. Most studies have identified coffee consumption as a protective factor against melanoma. For example, an observational study reported that consuming one cup of coffee per day was associated with a 54% reduction in melanoma risk.^[^ ^305^ ^]^ Similarly, Wu et al. found that higher caffeine intake was associated with a lower risk of melanoma. Specifically, individuals consuming more than 393 mg of caffeine per day had a 22% reduced risk compared to those consuming less than 60 mg.^[^ ^306^ ^]^ Notably, decaffeinated coffee did not show a significant association with melanoma incidence. In a large cohort study involving over 160 000 participants, those who consumed more than four cups of coffee daily had a significantly lower incidence of melanoma compared to non‐coffee drinkers (HR = 0.72).^[^ ^307^ ^]^ Moreover, the type of coffee appears to influence its protective effects. Filtered coffee was associated with the reduced risk of melanoma, while instant and boiled coffee showed no significant association across different consumption levels.^[^ ^308^ ^]^ A meta‐analysis further supported these findings, suggesting that caffeinated—but not decaffeinated—coffee may have chemopreventive properties against malignant melanoma.^[^ ^309^ ^]^ However, there is a scarcity of research investigating the impact of coffee on the progression and mortality of melanoma, suggesting that a larger prospective studies and intervention studies are warranted.

**Figure 5:**
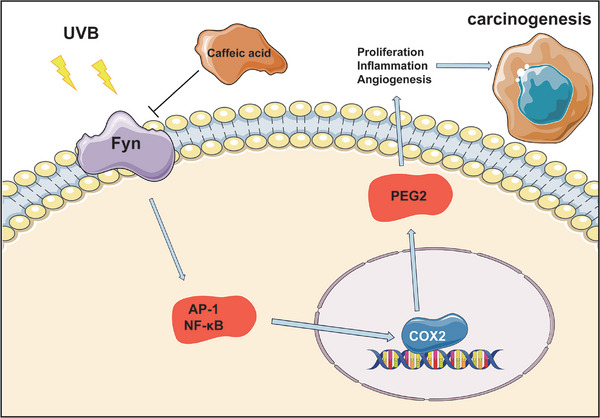
Proposed mechanism of caffeic acid in inhibiting UV‐induced melanoma progression. Caffeic acid inhibits UVB‐induced COX‐2 expression and PEG2 generation by blocking Fyn kinase activity, which occurs through the inhibition of the AP‐1 and NF‐κB pathways, thereby generating a skin‐protective effect.

The molecular mechanisms by which coffee consumption exerts its protective effects against melanoma development remain incompletely understood. Caffeine may inhibit melanoma cell proliferation by targeting tyrosine kinases, thereby reducing the secretion of pro‐inflammatory cytokines such as IL‐1β, CXCL10, MIP‐1α, and RANTES.^[^ ^310^ ^]^ Additionally, one study demonstrated that caffeine selectively induces the depletion of reduced thiols and exerts pro‐apoptotic effects in melanoma cells.^[^ ^311^ ^]^ In the context of immunotherapy, caffeine has been shown to enhance the anti‐tumor efficacy of anti‐PD‐1 monoclonal antibodies by increasing the infiltration of CD4⁺ and CD8⁺ T cells into the tumor microenvironment and decreasing the number of Tregs.^[^ ^312^ ^]^ UVR activates several transcription factors, including NF‐κB,^[^ ^313^ ^]^ COX‐2,^[^ ^314^ ^]^ and subsequently, drive the transcription of oncogenic and inflammatory genes, contributing to the pathogenesis of melanoma and other skin cancers. Compounds found in coffee may counteract UV‐induced carcinogenesis. Moreover, caffeic acid, another bioactive compound in coffee, has been shown to inhibit UVB‐induced COX‐2 expression by blocking Fyn kinase activity, which occurs through the inhibition of the activator protein‐1(AP‐1) and NF‐κB pathways, thereby generating a protective effect (**Figure** **5**).^[^ ^315^ ^]^ Therefore, moderate consumption of caffeinated coffee—such as 1–2 cups d^−1^—may help reduce the risk of melanoma and related skin malignancies.

### 5.4 UVR

UVR exposure is the most well‐established environmental risk factor for melanoma. Sunlight, the primary source of UVR on Earth, consists of approximately 95% UVA (320–400 nm) and 5% UVB (290–320 nm), while UVC rays are effectively blocked by the stratospheric ozone layer.^[^ ^316^ ^]^ Globally, increasing exposure to UVR—driven by behaviors such as recreational sunbathing,^[^ ^251^ ^]^ indoor tanning,^[^ ^317^ ^]^ and ozone layer depletion^[^ ^318^ ^]^—has been identified as a major contributor to the rising incidence of melanoma.^[^ ^319^ ^]^ A 20‐year meta‐analysis revealed a significant relationship between UVR and melanoma risk in individuals with Fitzpatrick skin types I–IV, showing that sunburn increased melanoma risk (OR = 1.66). Geographic factors and the frequency of outdoor activities were also identified as contributors.^[^ ^320^ ^]^ Among Medicare beneficiaries, the presence of actinic keratoses—a clinical marker of chronic UV damage—was associated with a 28.5% absolute 5‐year risk of developing skin cancer, including melanoma.^[^ ^321^ ^]^ Additionally, research into male‐pattern baldness revealed a higher incidence of head and neck melanoma in balding individuals, likely due to increased UV exposure in these areas. This relationship was further linked to genetic polymorphisms in pigmentation‐related pathways.^[^ ^322^ ^]^ Although UVR exposure is traditionally viewed as a key driver of cutaneous melanoma, alternative explanations such as increased diagnostic scrutiny have been proposed. For instance, a U.S. county‐level analysis showed that areas with greater economic and healthcare resources reported higher melanoma incidence, while daily UV dose was not correlated with incidence—suggesting reproducibility in diagnostic scrutiny's impact.^[^ ^323^ ^]^ Sun protection is highly recommended for patients with a history of melanoma. A study involving 448 male and 341 female melanoma patients found that inadequate sunscreen use more than doubled the risk of developing a second primary melanoma within two years (HR = 2.45).^[^ ^324^ ^]^ However, sun protection behaviors are influenced by various social determinants, particularly education level and socioeconomic status.

At the genetic level, researchers have identified additional UV‐induced substitutions, such as AC > TT and A > T, which can lead to the oncogenic *BRAF V600K* and *V600E* mutations, respectively.^[^ ^325^ ^]^ Besides, hotspot mutations enriched in UV mutational signatures disrupt amino acid sequences essential for the binding of SH3 domain‐containing proteins, which play a critical role in p53 function. These protein–protein interaction sites include MDM2, a negative regulator of p53; XAF1, which enhances p53‐mediated apoptosis; and PIN1, a proline isomerase necessary for the proper structural folding of the p53 domain. They are strongly associated with melanoma and other skin cancers, highlighting their role as driver mutations.^[^ ^326^ ^]^ Beyond these, transcription is also vital for maintaining genome integrity. UVR promotes the assembly of RNA polymerase II pre‐initiation complexes, thereby enhancing transcription‐coupled DNA repair activity within two hours of exposure. This rapid repair response reduces the risk of mutagenesis and contributes to the unique mutational spectrum observed in melanoma.^[^ ^327^ ^]^ Moreover, UV exposure can alter the DNA‐binding specificity of transcription factors, increasing their affinity for non‐consensus sequences. This shift disrupts transcriptional networks and competes with DNA repair mechanisms at sites of damage.^[^ ^328^ ^]^

In addition to promoting mutagenesis, UVR also alters the skin microenvironment, which likely contributes to the initiation and progression of melanoma. Within the epidermis, each melanocyte interacts with ≈30–40 neighboring keratinocytes through long dendritic processes.^[^ ^329^ ^]^ Cross‐talk between keratinocytes and melanocytes—as well as with other skin‐resident cells—facilitates adaptation to external stimuli. Upon UVR exposure, surviving keratinocytes secrete melanocyte growth including α‐melanocyte‐stimulating hormone (α‐MSH) and Endothelin‐1, which stimulate both cytokine and melanin production.^[^ ^330^ ^]^ In addition, direct and indirect interactions between melanoma cells and surrounding keratinocytes or fibroblasts can influence melanoma cell behavior and metastatic potential.^[^ ^331^, ^332^ ^]^ Concurrently, UVR modulates the activity of immune cells within the TME. Chronic UV exposure suppresses CD8⁺ T cell cytotoxicity in skin‐draining lymph nodes by promoting a Ly6a^high^ T cell subpopulation, thereby inducing immune suppression and resistance to anti‐PD‐1 immunotherapy. Notably, treatment with anti‐Ly6a antibodies enhances antitumor activity by reprogramming mitochondrial metabolism through the ERK/c‐Myc signaling axis.^[^ ^333^ ^]^ In addition, IL‐15, a cytokine upregulated in skin cells following UVR, exhibits sustained expression during melanoma progression. Analyses of public datasets have shown that elevated levels of soluble IL‐15/IL‐15Rα are associated with impaired NK cell functionality in late‐stage melanoma, while high expression correlates with improved survival in stages II and III.^[^ ^334^ ^]^

Although both UVA and UVB exposure increase the risk of basal cell carcinoma, squamous cell carcinoma, and melanoma through different mechanisms, these two aspects are rarely discussed separately in practice. Historically, the mutagenic effects of UVB—primarily resulting from its direct DNA damage through the formation of cyclobutane pyrimidine dimers (CPDs) and 6‐4 photoproducts—have been considered more significant. However, UVA, previously believed to have a weaker association with melanoma initiation, has recently received increased attention. Studies have revealed that UVA penetrates into the dermis and causes damage to DNA and other cellular and extracellular components primarily through the generation of ROS.^[^ ^335^ ^]^ ROS play dual roles in melanoma progression, functioning both as promoters of tumor growth and inducers of apoptosis.^[^ ^334^, ^336^ ^]^ Elevated ROS levels can accelerate tumor development by activating signaling pathways associated with increased cell proliferation, such as the PI3K/Akt and MAPK pathways.^[^ ^337^ ^]^ ROS can also trigger the PI3K/Akt cascade by inactivating phosphatase and tensin homolog (PTEN), a negative regulator of this pathway.^[^ ^338^ ^]^ This creates a vicious cycle wherein Akt activation leads to further increases in cellular ROS levels, thereby promoting the survival and proliferation of melanoma cells.^[^ ^339^ ^]^ To combat oxidative stress, tumor cells activate the Nuclear factor erythroid 2–related factor 2 (Nrf2) signaling pathway,^[^ ^340^ ^]^ enhancing their antioxidant capacity and enabling resistance to therapeutic interventions.^[^ ^341^ ^]^ Moreover, Nrf2 has recently been implicated in the development of resistance to targeted therapies. Specifically, Nrf2 has been identified as a critical factor contributing to resistance against BRAF and MEK inhibitors in melanoma cells.^[^ ^342^ ^]^

In recent years, a large number of novel materials and pharmacological agents have been developed for photoprotection, aiming not only to enhance UV‐blocking efficiency but also to prevent UV‐induced genetic damage. Among them, acetyl zingerone and its derivatives have emerged as potent inhibitors of both immediate and delayed formation of CPDs following UV exposure. These compounds promote DNA repair by upregulating key nucleotide excision repair genes, such as Xpa and Xpc, without exhibiting cytotoxicity in skin cells—highlighting their potential for both sunscreen formulation and clinical application.^[^ ^343^ ^]^ Additionally, l‐Cysteine‐derived carbon dots (GLCDs) exhibit remarkable broad‐spectrum UV absorption across the 200–400 nm range, even at low concentrations (0.5 mg mL^−1^). GLCDs have been shown to alleviate UVB‐induced oxidative stress and attenuate skin aging by enhancing collagen expression.^[^ ^343^ ^]^ In summary, UV exposure represents a critical but modifiable risk factor for melanoma, driving its pathogenesis via DNA damage and immune suppression. Nevertheless, the interplay between UV radiation and other environmental or lifestyle factors remains insufficiently characterized. Future studies should aim to elucidate these complex interactions to uncover potential synergistic mechanisms and identify novel targets for melanoma prevention.

### 5.5 Circadian Rhythms

Circadian rhythms coordinate the behavior and physiology of all organs to maintain whole‐body homeostasis.^[^ ^344^ ^]^ While acute disruptions can lead to transient discomfort, long‐term irregularities in the circadian rhythm may increase the risk of numerous diseases, particularly cancers.^[^ ^345^ ^]^

Night shift work is associated with increased risk of several cancers, but the risk of melanoma among night shift workers is counterintuitive. In the Nurses' Health Study involving 68 336 women, working ≥10 years of rotating night shifts was paradoxically associated with a 44% decreased risk of melanoma.^[^ ^346^ ^]^ Similarly, short sleep duration (≤6 h d^−1^) was linked to lower risk of melanoma in female (HR = 0.68, 95% CI: 0.46–0.98).^[^ ^347^ ^]^ However, another study found no significant increase in melanoma risk among male shift workers,^[^ ^348^ ^]^ while a recent dose–response analysis reported a 2% annual increase in melanoma risk with each year of shift work.^[^ ^349^ ^]^ These discrepancies warrant further investigation into underlying biological mechanisms.

In mammals, the suprachiasmatic nucleus (SCN), serving as the central pacemaker, governs and synchronizes circadian autonomy through a molecular mechanism based on transcriptional and translational feedback loops. The core transcription factor Bmal1 (brain and muscle ARNT‐like 1)/CLOCK (circadian locomotor output cycles kaput) heterodimerizes and activates the transcription of target genes, including the Period (Per1, Per2) and Cryptochrome (Cry1, Cry2) genes, by binding to E‐box elements in their promoters.^[^ ^350^, ^351^ ^]^ As PER and CRY proteins accumulate in the cytoplasm, they form complexes that translocate into the nucleus and, in turn, inhibit Bmal1/CLOCK activity, thereby repressing their own transcription. This negative feedback results in a decline in PER and CRY levels, ultimately initiating a new cycle. Additional loops involving nuclear receptors such as REV‐ERBα/β and RORα/β/γ further regulate the expression of Bmal1, providing robustness and precision.^[^ ^352^ ^]^ Post‐translational modifications, including phosphorylation by kinases like casein kinase 1δ/ε, also play a critical role in fine‐tuning the timing and stability of clock proteins. The interconnected and fine‐tuned molecular oscillators are not only restricted to the SCN but also present in the peripheral tissues, such as the liver, immune system, and skin, where they regulate tissue‐specific circadian physiology and are integrated with whole‐body physiological demands.^[^ ^353^, ^354^ ^]^

Circadian disruption in melanocytes and melanoma cells may compromise DNA repair capacity, increasing susceptibility to UV‐induced mutagenesis. BMAL1/CLOCK heterodimers have been shown to enhance nucleotide excision repair (NER) efficiency by promoting XPA expression, thereby potentially mitigating UVR‐induced DNA damage in skin.^[^ ^355^, ^356^ ^]^ In melanocytes, BMAL1 is found to bind the promoter region of MITF rhythmically, driving its daytime expression and coordinating melanogenesis.^[^ ^357^ ^]^ This suggests a potential among between circadian clock activity, melanocyte homeostasis, and UV protection. While direct evidence connecting circadian regulators to DNA repair in melanoma remains limited, circadian transcription factors have been implicated in modulating key DNA repair genes—such as BRCA1 and RAD50—in other cancers, including breast cancer,^[^ ^358^ ^]^ colorectal cancer,^[^ ^359^ ^]^ and hepatocellular carcinoma.^[^ ^360^ ^]^ These findings support the hypothesis that the circadian clock may influence melanoma pathogenesis via regulation of DNA repair pathways, though further mechanistic studies are needed to define this axis more precisely in melanoma.

Melanoma cells exhibit altered circadian gene expression. Bmal1 was shown to exert cell state‐dependent effects on melanoma immunity, tumorigenicity, and metastasis. On the one hand, loss of Bmal1 in murine melanoma models disrupted circadian rhythmicity, suppressed hypoxia‐related gene expression, and inhibited tumor growth. On the other hand, overexpression of Bmal1 enhanced melanoma cell plasticity by promoting a transition from a Sox10^high^ proliferative state to a Sox9^high^ mesenchymal‐like, immune‐tolerant state. This phenotypic switch was mediated through a non‐canonical interaction between Bmal1 and non‐muscle myosin heavy chain IIA (Myh9), which activated the MRTF‐SRF signaling pathway and increased AP‐1 transcriptional activity.^[^ ^361^ ^]^ Nevertheless, BMAL1 has been revealed as a clinically relevant prognostic factor and biomarker for T‐cell‐based immunotherapies for melanoma patients,^[^ ^362^ ^]^ suggesting a complex role of circadian genes in tumor immunity and progression.

Emerging research highlights that immune surveillance exhibits circadian oscillation, which has important implications for the efficacy of immunotherapy. Circadian disruption could induce the loss or inversion of daily patterns of M1 (proinflammatory) and M2 (anti‐inflammatory) macrophages and cytokine levels in spleen and tumor tissues, and therefore facilitate tumor growth.^[^ ^363^ ^]^ Both meta‐analyses and retrospective studies in patients with melanoma found that morning administration of ICIs significantly prolonged OS compared to afternoon or evening treatment.^[^ ^364^, ^365^, ^366^ ^]^ Experimental studies further support this by showing circadian oscillations in both the abundance and phenotypes of TILs.^[^ ^367^, ^368^ ^]^ First, the expression of Pdcd1 (coding PD‐1) peaks at zeitgeber time 1 (ZT1, corresponding to the morning in mice, opposite to humans) in CD8^+^ effector T cells. And CD8^+^ T cells exhibit a more suppressed phenotype in ZT13 (evening) and a more anti‐tumorigenic signature in ZT1 (morning).^[^ ^369^ ^]^ The circadian regulation of co‐stimulatory molecule CD80 expression and rhythmic migration of DCs to TDLNs collectively drive the time‐of‐day–dependent priming of tumor‐specific CD8⁺ T cell responses.^[^ ^370^ ^]^ As gatekeepers at the blood‐tumor interphase, endothelial cells also contribute, exhibiting higher intercellular adhesion molecule 1 (ICAM‐1) expression in ZT13 than in ZT1, thereby modulating leukocyte infiltration.^[^ ^369^ ^]^ Several circadian clock genes also underscore this interplay. DEC2 exhibits circadian oscillation in TAMs, with elevated expression coinciding with the trough of Pdcd1 mRNA levels. It functions as a repressor by periodically inhibiting NF‐κB‐mediated transactivation of Pdcd1, thereby contributing to the rhythmic expression of PD‐1.^[^ ^367^ ^]^ RORα (RORA) binds to the CD274 promoter and forms an inhibitory complex with HDAC3 to suppress PD‐L1 expression, therefore enhancing T cell recruitment and cytotoxicity.^[^ ^371^, ^372^ ^]^

Circadian regulation is essential for maintaining physiological homeostasis. However, the mechanisms by which circadian disruption affects skin homeostasis and DNA repair—thereby contributing to melanocyte transformation, melanoma progression, and metastasis—remain to be fully elucidated. With growing clinical evidence, circadian‐informed strategies may help refine therapeutic decision‐making and improve cancer treatment outcomes.

Current evidence indicates that multiple behavioral factors—including smoking, alcohol consumption, caffeine intake, UVR exposure, and circadian disruption—collectively influence melanoma pathogenesis through complex, interrelated mechanisms (Table S4, Supporting Information). Smoking presents a paradoxical inverse association with melanoma incidence, potentially mediated by nicotine‐induced immunosuppression or activation of the Wnt/β‐catenin pathway; however, it clearly contributes to tumor progression and increased mortality, warranting individualized risk–benefit assessments. Alcohol exerts carcinogenic effects primarily through its metabolite acetaldehyde, which impairs DNA repair mechanisms and suppresses immune responses. In contrast, caffeine demonstrates protective effects by inhibiting NF‐κB signaling and enhancing the efficacy of anti‐PD‐1 immunotherapy, supporting its potential role in melanoma prevention. UVR remains the primary environmental driver of melanoma, inducing direct DNA damage (e.g., *BRAF* mutations), generating ROS, and promoting immunosuppression. Notably, synergistic interactions between UVR and alcohol‐related DNA repair deficiencies may further amplify risk. Circadian disruption also contributes to melanoma pathogenesis by altering immune cell trafficking and modulating tumor immunity via dysregulation of core clock genes, thereby positioning chronotherapy and circadian‐aligned immunotherapy as promising avenues for clinical intervention. These insights underscore the importance of integrated behavioral strategies—encompassing smoking cessation, alcohol moderation, UV protection, and circadian rhythm maintenance—that consider not only individual risk factors but also their potential interactions.

Future investigations should explore circadian modulators such as melatonin, which both attenuate UVR‐induced damage^[^ ^373^ ^]^ and exhibit intrinsic antitumor properties.^[^ ^374^ ^]^ In parallel, machine learning‐based approaches may offer a powerful framework for quantifying these multifactorial behavioral influences and constructing predictive models tailored to individual melanoma risk. This integrative perspective highlights the imperative for translational research bridging behavioral epidemiology with precision medicine in melanoma prevention and therapy.

## 6 Concluding Remarks and Future Perspectives

In recent years, significant advances in melanoma research have spanned from more scientifically grounded preventive strategies to the development of sophisticated clinical screening technologies, markedly improving early detection and intervention. However, the diversity and complexity of lifestyle and host‐related factors continue to pose substantial challenges in understanding melanoma pathogenesis and optimizing therapeutic strategies. Host factors such as sex and age exert multifaceted influences on melanoma through genetic, hormonal, and immunological mechanisms. Males exhibit higher rates of *BRAF* and *NRAS* mutations and lower expression of ERβ, whereas older individuals tend to show increased *NRAS* but reduced *BRAF* mutations, indicating distinct oncogenic drivers—particularly in high‐risk elderly males. Hormonally, estrogen exerts antitumor effects via the GPER/cAMP/MITF pathway, with elevated levels in females and obese males potentially contributing to improved prognosis. Immunologically, estrogen enhances antitumor immunity by promoting IL‐1β secretion through ERβ signaling, while aging is associated with immunosenescence; nonetheless, a preserved CD8⁺ T cell/Treg ratio in elderly individuals may support responsiveness to immunotherapy. Pharmacological factors also influence melanoma outcomes. High‐dose GC administration impairs CD8⁺ TIL activity and activates the ROCK1/2–PI3K/Akt axis, thereby facilitating metastasis. Antihistamines, through antagonism of the HRH1, suppress M2 macrophage polarization, whereas HRH4 agonists exhibit emerging antitumor properties. NSAIDs confer greater therapeutic benefits in female patients. Metformin promotes apoptosis via modulation of microRNAs and the AMPK/PRODH/POX pathway, while statins demonstrate dual effects—enhancing NK cell cytotoxicity while potentially expanding Tregs. Metabolic factors—notably obesity—exert immunosuppressive effects through adipokine signaling and metabolic dysregulation. BMI, although commonly used, insufficiently captures adiposity‐related risks. Interestingly, a BMI >35 kg m^−2^ appears to enhance immunotherapy response in males but not females, underscoring sex‐specific metabolic‐immune interactions. In parallel, HFD and PA modulate tumor progression via adipose tissue remodeling and nutrient competition with tumor cells. Behavioral and environmental factors further shape melanoma risk and prognosis. Smoking shows a paradoxical inverse association with melanoma incidence—possibly due to nicotine‐induced immunosuppression or Wnt/β‐catenin activation—but unequivocally worsens disease outcomes. Alcohol promotes carcinogenesis through its metabolite acetaldehyde, which impairs DNA repair and immune function. Conversely, caffeine exerts protective effects by inhibiting NF‐κB signaling and augmenting anti–PD‐1 therapy efficacy. UVR remains a principal environmental driver, inducing DNA damage (e.g., *BRAF* mutations), generating ROS, and suppressing immunity. Circadian disruption, by altering immune cell trafficking and clock gene expression, may further exacerbate melanoma progression, highlighting the potential of circadian‐aligned (chrono‐optimized) therapeutic approaches.

### 6.1 Take Home Message

This review highlights the critical role of lifestyle factors in melanoma, hoping to inspire greater attention to the impact of lifestyle factors on melanoma risk assessment and management. Therefore, we concluded our insights and advice for researchers and clinicians:

- Age‐stratified research should move beyond binary classifications by integrating machine learning and aging biomarkers to refine prognostic models.
- Sex–age interactions call for multi‐omics approaches to dissect mechanisms such as fibroblast‐mediated AXL/BMP2 signaling, especially in elderly males.
- Drug response assessments must account for indication‐specific effects (e.g., statins in hyperlipidemia) and sex differences (e.g., NSAIDs), while seeking safer alternatives to glucocorticoids (e.g., JAKi, pending validation).
- Obesity metrics should align with underlying biology (e.g., visceral adiposity index) to resolve contradictions like the “obesity paradox,” and lifestyle studies should consider obesity as a metabolic hub.
- Behavioral and circadian factors influence treatment efficacy and require rigorous control of confounders. Comparative studies (e.g., melatonin vs circadian interventions) may clarify mechanisms. Time‐of‐day–dependent variation in ICI response underscores the potential of chrono‐immunotherapy strategies that integrate behavioral interventions.

Together, these innovations promise to transform lifestyle research into actionable strategies for melanoma prevention and care.

## Conflict of Interest

The authors declare no conflict of interest.

## Author Contributions

H.W., H.Z., and Y.Z. contributed equally to this work. W.G. and C.L. considered and designed the review. H.W. wrote the main manuscript text, H.Z. and Y.Z. prepared figures and revised the manuscript. All authors read and approved the final manuscript.

## Supporting information



Supporting Information

Supporting Information

Supporting Information

Supporting Information
