# Supplementary material for: Life Factors and Melanoma: From the Macroscopic State to the Molecular Mechanism
Source: Adv Sci (Weinh). 2025 Oct 14;12(43):e01388. doi: 10.1002/advs.202501388 (PMC12631907; doi:10.1002/advs.202501388)
Supplement: Supplementary file 1 — Supporting Information [file ADVS-12-e01388-s002.docx]

Supplemented Table.1 Graphical summary of intrinsic host characteristics

| Category | ​Pathway/Mechanism | ​Groups | ​Effect | ​Supporting Evidence |
| --- | --- | --- | --- | --- |
| ​Gender-Related Pathways |  |  |  |  |
| Genetic Mutation Differences | Higher number of missense mutations (e.g., BRAF/NRAS) | Male vs. Female | ↑ Risk | Higher mutational burden in males correlates with worse survival (Refs. 28, 29) |
| Estrogen Receptor Signaling | ERβ activation upregulates IL-1β, triggering antitumor neutrophil chemotaxis; inhibits EMT | Female (high ERβ expression) | ↓ Protective | ERβ expression increases in acidic conditions in females (Ref. 37); higher estrogen in obese males may improve prognosis (Ref. 38) |
| GPER Pathway | GPER activation reduces melanoma cell proliferation and promotes melanogenesis | Female (high GPER activity) | ↓ Protective | GPER inhibits tumor via cAMP/PKA/MITF pathway (Ref. 32) |
| ERα vs. ERβ Balance | ERα promotes tumor proliferation, while ERβ exerts antitumor effects (antagonizes ERα) | Male (ERα dominance) | ↑ Risk | High ERβ expression correlates with better prognosis (Refs. 33, 34) |
| ​Age-Related Pathways |  |  |  |  |
| Mutation Spectrum Differences | ↑ NRAS mutations (poor prognosis) in elderly; ↓ BRAF mutations; young patients show ↑ BRAF V600E mutations | Elderly vs. Young | ↑ Risk | NRAS mutations increase with age (Refs. 67, 70-71); BRAF V600E more common in young patients (Refs.70, 72, 75) |
| Microenvironment Alterations | Aged fibroblasts secrete ↑ sFRP2 → suppresses β-catenin/MITF, increasing resistance to targeted therapy | Elderly | ↑ Risk | sFRP2 promotes angiogenesis and metastasis via Wnt antagonism (Ref. 78) |
| HAPLN1 Reduction | ↓ HAPLN1 in aged skin → reduced CD4^+^/CD8^+^ T-cell infiltration, ↑ risk of lymphatic metastasis | Elderly | ↑ Risk | HAPLN1 loss promotes distant metastasis (Refs. 76-77) |
| Immunosenescence | ↑ CD8^+^ T-cell/Treg ratio in elderly may enhance ICI efficacy | Elderly (partial benefit) | ↓ Protective | Elderly patients show comparable or better ICI response (Refs. 57-58) |
| ​Interactive Pathways |  |  |  |  |
| Gender-Age Interaction | Male aging drives immunosuppressive TME via AXL/BMP2 signaling; female aging retains ERβ activity, partially offsetting risk | Elderly Males vs. Elderly Females | Males: ↑ Risk | Latest research highlights sex-specific aging TME differences (Ref. 82) |
