# Supplementary material for: Life Factors and Melanoma: From the Macroscopic State to the Molecular Mechanism
Source: Adv Sci (Weinh). 2025 Oct 14;12(43):e01388. doi: 10.1002/advs.202501388 (PMC12631907; doi:10.1002/advs.202501388)
Supplement: Supplementary file 2 — Supporting Information [file ADVS-12-e01388-s003.docx]

Supplemented Table.2 Graphical summary of pharmacological influences

| Medication | ​Pathway/Mechanism | ​Effect | ​Supporting Evidence |
| --- | --- | --- | --- |
| ​Glucocorticoids | Suppress CD8^+^ TIL activity and tumor-killing capacity | ↑Risk | High-dose GCs (≥60mg prednisone equivalent) associated with a 126% increase in all-cause mortality within 3 months post-ICI (Ref. 89) |
|  | Activate ROCK1/2-PI3K/Akt pathway to promote metastasis |  | *In vitro*: Dexamethasone impairs CD8^+^ TIL function (Ref. 96) |
| ​JAK Inhibitors | Modulate T-cell function (e.g., tofacitinib) | ↓Protective | Preclinical studies show enhanced anti-PD-1 efficacy (Refs. 93-94) |
|  | Dynamically regulate myeloid cell differentiation |  |  |
| ​Antihistamines | Block HRH1 receptor, inhibiting M2-like macrophage polarization | ↓Protective | HRH1 knockout or antihistamine treatment restores T-cell function (Ref. 99) |
|  | HRH4 agonists promote antitumor immunity |  | HRH4 agonists show antitumor effects in animal models (Ref. 102) |
| ​NSAIDs | Inhibit COX-1/2 → Reduce PGE2 → Suppress platelet aggregation and pre-metastatic niche formation | ↓ (more pronounced in women) | Long-term aspirin use reduces melanoma risk by 30% in women (Ref.109) |
|  | Downregulate AXL/PKA/NF-κB pathways |  | Post-diagnostic NSAID use linked to lower mortality in stage II/III patients (Ref. 114) |
| ​Metformin | Activate AMPK/PRODH/POX → Induce ROS-mediated apoptosis | ↓Protective | Diabetic patients on metformin show reduced 5-year recurrence (Ref. 131) |
|  | Promote CD8^+^ T-cell infiltration and M1-TAM polarization |  | Enhances NK cell antitumor activity in mouse models (Ref. 138) |
| ​Statins | Inhibit RhoA pathway → Suppress metastasis | (controversial) | Statin users have higher 5-year survival rates (Ref. 141) |
|  | Upregulate MICA to enhance NK cell cytotoxicity |  | Synergistic effect with dacarbazine in mice (Ref. 151) |
|  | Increase Treg proportion via Foxp3 mRNA transcription |  | Controversy: Some studies suggest increased risk (Ref. 157) |
| ​PCSK9 Inhibitors | Block PCSK9-LDLR interaction → Enhance T-cell cholesterol uptake and function | ↓Protective | Mendelian randomization suggests PCSK9 inhibitors may be protective (Ref. 144) |
| ​Antibiotics | Disrupt gut microbiota → Impair DC and CD8^+^ T-cell responses | ↑Risk | Antibiotic use within 60 days pre-ICI correlates with worse PFS/OS (Ref. 158) |
|  |  |  | Dysbiosis promotes tumor progression via reduced T-cell activation (Ref. 165) |
