# Supplementary material for: Life Factors and Melanoma: From the Macroscopic State to the Molecular Mechanism
Source: Adv Sci (Weinh). 2025 Oct 14;12(43):e01388. doi: 10.1002/advs.202501388 (PMC12631907; doi:10.1002/advs.202501388)
Supplement: Supplementary file 3 — Supporting Information [file ADVS-12-e01388-s004.docx]

Supplemented Table.3 Graphical summary of metabolic conditions

| Metabolic Conditions | ​Pathway/Mechanism | ​Effect | ​Supporting Evidence |
| --- | --- | --- | --- |
| ​Obesity | Insulin resistance activates PI3K/Akt pathway, reducing efficacy of chemotherapy/targeted therapy. | ↑Risk | "Increased insulin levels dampen the therapeutic efficacy of dacarbazine and PLX4720 (mutant BRAF inhibitor) in melanoma cells via activation of the PI3K/Akt pathway." (Ref. 200) |
|  | Adipocytes secrete CCL2, M-CSF, and CCR7, promoting M2-TAMs, angiogenesis, and lymphangiogenesis. | ↑Risk | "Mature adipocytes enhance the expression of CCL2, M-CSF, and CCR7 mRNAs in melanoma cells, increasing M2-TAMs and VEGF-D-driven angiogenesis." (Ref. 204) |
|  | Leptin activates OB-Rb receptor, stimulating Akt/ERK and promoting tumor proliferation/drug resistance. | ↑Risk | "Leptin interacts with OB-Rb on melanoma cells, increasing Akt/ERK activation and protecting cells against drugs." (Ref. 210) |
|  | Adipocyte-derived extracellular vesicles (Ad-EV) transfer β-catenin, suppressing CDKN2A and enhancing metastasis. | ↑Risk | "Ad-EV conveys β-catenin to melanoma cells, decreasing p16INK4A and increasing motility." (Ref. 203) |
|  | Obesity induces CD8^+^ T-cell exhaustion (↑PD-1, Tim3, Lag3) and impairs NK cell function. | ↑Risk | "Diet-induced obesity (DIO) mice showed increased exhausted CD8^+^ TILs and lipid-accumulated NK cells with impaired cytotoxicity." (Ref 214-216) |
|  | ​"Obesity paradox": Higher BMI may improve immunotherapy (ICI) response. | ↓Protective | "Overweight/obese patients may experience survival benefits with combination immunotherapy." (Ref 186) |
| Diets & nutrients |  |  |  |
| ​Caloric Restriction (CR) | Suppresses NF-κB, preserves E-cadherin, and reduces angiogenesis/metastasis. | ↓Protective | "CR reduces micro-vessel density and inhibits melanoma growth via NF-κB suppression and ECM remodeling." (Ref. 221) |
|  | Chronic CR leads to sarcopenia, impairing CD8^+^ T-cell immune surveillance. | ↑Risk | "Patients with sarcopenia may have poorer immunotherapy responses due to reduced T-cell infiltration and activation." (Refs. 222-223) |
| ​Intermittent Fasting | Enhances chemotherapy (e.g., Adriamycin) and targeted therapy (e.g., sorafenib) sensitivity. | ↓Protective | "Cyclic fasting increased melanoma susceptibility to Adriamycin in mice." (Ref. 229) |
|  | Synergizes with anti-PD-L1/OX40, improving tumor control and reducing ICI cardiotoxicity. | ↓Risk | "Fasting mimicking diets enhance ICI efficacy and reverse cardiac fibrosis in murine models." (Ref. 231) |
| ​Mediterranean Diet (MD) | High n-3 fatty acids and vegetable intake reduce melanoma risk. | ↓Protective | "Fish, vegetables, and olive oil in MD correlate with lower melanoma incidence." (Refs. 232-233) |
|  | Dietary fiber modulates gut microbiota, increasing SCFAs and improving immunotherapy PFS. | ↓Protective | "Higher fiber intake correlates with improved PFS in melanoma patients on ICB therapy." (Ref.235) |
|  | High blood SCFAs may suppress anti-CTLA-4 efficacy (↑Tregs). | ↑Risk | "Butyrate inhibits anti-CTLA-4-induced T-cell activation and memory responses." (Ref. 240) |
| ​High-Fat Diet | Promotes IL-6/JAK2/osteopontin activation via bone marrow adipocytes, accelerating metastasis. | ↑Risk | "HFD increases bone marrow adipocytes and IL-6-driven tumor cell activation." (Ref. 241) |
|  | High ω-3/ω-6 intake associated with thicker melanomas (>2 mm). | ↑Risk | "Australian study linked high omega-3/6 intake to thicker melanomas." (Ref. 242) |
| ​Exercise | Induces NK cell (epinephrine/IL-6-dependent) and CD8^+^ T-cell tumor infiltration. | ↓Protective | "Exercise mobilizes NK cells into circulation and redistributes them to tumors." (Ref. 259) |
|  | Metabolic competition restricts tumor nutrient supply. | ↓Protective | "Exercise-induced metabolic reprogramming forms a nutrient barrier around tumors." (Ref. 224) |
|  | Outdoor exercise may increase UV exposure risk (controversial). | ↑Risk | "High leisure-time physical activity was associated with elevated melanoma risk (HR 1.27)." (Ref. 261) |
