# Supplementary material for: Life Factors and Melanoma: From the Macroscopic State to the Molecular Mechanism
Source: Adv Sci (Weinh). 2025 Oct 14;12(43):e01388. doi: 10.1002/advs.202501388 (PMC12631907; doi:10.1002/advs.202501388)
Supplement: Supplementary file 4 — Supporting Information [file ADVS-12-e01388-s001.docx]

Supplemented Table.4 Graphical summary of behavioral risk profiles

| ​Behavioral Factor | ​Pathway/Mechanism | ​Effect | ​Supporting Evidence |
| --- | --- | --- | --- |
| ​Smoking | Immunosuppressive effect reduces UVR-induced inflammation | ↓ Protective | "Tobacco contains very high lithium levels... enhancing the β-catenin activity" (Ref. 281) |
|  | Lithium inhibits GSK-3β, activating β-catenin pathway |  | "Immunosuppressive effect... protects melanocytes from UVR-induced inflammation" (Refs. 271, 280) |
|  | Survival bias (high overall mortality masks melanoma death) |  | "High mortality may lead to survival bias" (Ref. 277) |
|  | Nicotine activates Akt/ERK pathway via α9-nAChR, promoting PD-L1 expression and EMT | ↑ Risk | "Nicotine induces... Alpha-9 nicotinic acetylcholine receptor activation, promoting PD-L1 expression via STAT3" (Ref. 287) |
|  |  |  | "Smokers had thicker tumors and more ulcerated areas" (Refs. 296, 298) |
| ​Alcohol Consumption | Acetaldehyde causes DNA damage | ↑ Risk | "AcAH causes DNA damage and blocks repair" (Ref. 301) |
|  | Suppresses memory T cells and NK cell function |  | "Chronic alcohol inhibits memory T cells and CD62L^+^ NK cells" (Refs. 296, 298) |
|  | Upregulates VEGF to promote angiogenesis |  | "Ethanol up-regulates VEGF (2.16-fold tumor weight increase)" (Ref. 299) |
| ​Coffee/Caffeine Intake | Inhibits tyrosine kinases and inflammatory cytokines (e.g., IL-1β) | ↓ Protective | "Caffeine reduces IL-1β, IP-10 secretion" (Ref. 310) |
|  | Blocks UV-induced NF-κB/p38 MAPK pathway |  | "Inhibits UV-induced NF-κB by interfering with ATM/PKC" (Ref. 326) |
| ​UV Radiation (UVR) | Direct DNA damage (CPD/6-4 photoproducts) | ↑ Incidence | "UV induces BRAF V600E/K mutations" (Ref. 326) |
|  | ROS activates PI3K/Akt/MAPK pathways |  | "ROS activate PI3K/Akt via PTEN inhibition" (Ref. 338) |
|  | Immune suppression (inhibits CD8^+^ T cells) |  | "UVR upregulates Ly6a high T-cells, suppressing CD8^+^ cytotoxicity" (Ref. 334) |
| ​Circadian rhythms(disruption) | BMAL1 suppresses melanoma growth via non-canonical Myh9-MRTF/SRF pathway | ↓ (controversial) | "BMAL1 loss suppresses hypoxia genes; overexpression promotes immune-tolerant state" (Ref. 362) |
|  |  |  | "Morning ICI administration improves OS (HR=0.64)" (Refs. 365, 367) |
|  | Circadian misalignment impairs immune surveillance (disrupted rhythmic infiltration of DCs/CD8^+^ T cells) | ↑ Risk | "Circadian disruption inverts M1/M2 macrophage patterns" (Ref. 363) |
|  |  |  | "DEC2 oscillates to rhythmically inhibit PD-1 in TAMs" (Ref. 368) |
